# Promising FDA-approved drugs with efflux pump inhibitory activities against clinical isolates of *Staphylococcus aureus*

**DOI:** 10.1371/journal.pone.0272417

**Published:** 2022-07-29

**Authors:** Safaa Abdel-Aal Mohamed Abdel-Karim, Amira Mohamed Ali El-Ganiny, Mona Abdelmonem El-Sayed, Hisham Abdelmonem Abdelhamid Abbas

**Affiliations:** Department of Microbiology and Immunology, Faculty of Pharmacy, Zagazig University, Zagazig, Egypt; Indiana University School of Medicine-Northwest, UNITED STATES

## Abstract

**Background and objectives:**

*Staphylococcus aureus* is an opportunistic pathogen that causes wide range of nosocomial and community-acquired infections which have spread worldwide leading to an urgent need for developing effective anti-staphylococcal agents. Efflux is an important resistance mechanism that bacteria used to fight the antimicrobial action. This study aimed to investigate the efflux mechanism in *S*. *aureus* and assess diclofenac, domperidone, glyceryl trinitrate and metformin as potential efflux pump inhibitors that can be used in combination with antibiotics for treating topical infections caused by *S*. *aureus*.

**Materials and methods:**

Efflux was detected qualitatively by the ethidium bromide Cart-Wheel method followed by investigating the presence of efflux genes by polymerase chain reaction. Twenty-six isolates were selected for further investigation of efflux by Cart-Wheel method in absence and presence of tested compounds followed by quantitative efflux assay. Furthermore, antibiotics minimum inhibitory concentrations in absence and presence of tested compounds were determined. The effects of tested drugs on expression levels of efflux genes *nor*A, *fex*A and *tet*K were determined by quantitative real time-polymerase chain reaction.

**Results:**

Efflux was found in 65.3% of isolates, the prevalence of *nor*A, *tet*K, *fex*A and *msr*A genes were 91.7%, 77.8%, 27.8% and 6.9%. Efflux assay revealed that tested drugs had potential efflux inhibitory activities, reduced the antibiotic’s MICs and significantly decreased the relative expression of efflux genes.

**Conclusion:**

Diclofenac sodium, domperidone and glyceryl trinitrate showed higher efflux inhibitory activities than verapamil and metformin. To our knowledge, this is the first report that shows that diclofenac sodium, glyceryl trinitrate and domperidone have efflux pump inhibitory activities against *S*. *aureus*.

## Introduction

*Staphylococcus aureus* is a commensal human bacterium that is widely spread in community and healthcare settings [1]. When *S*. *aureus* invades the host immune system, it crosses the epithelial barrier; gains access to deeper tissues as blood and other tissues and thus causes many complicated infections as skin and soft tissue infections, bacteremia, endocarditis, pneumonia, and osteomyelitis [2, 3]. Its nosocomial infections represent a serious problem as they cause high morbidities and mortalities [4]. In hospitals environment, *S*. *aureus* exposed to different antibiotics, disinfectants and sanitizers; therefore, it gained an enormous resistance and became multidrug-resistant (MDR) [4, 5]. In addition, the presence of resistance determinants on mobile genetic elements as transposons, integrons and plasmids has led to the wide spread of MDR strains [6–8].

*Staphylococcus aureus* exhibits diverse antibiotic resistance mechanisms, such as mutation of target site, antibiotics efflux systems, enzymatic inactivation of antibiotics and changes in the antibiotic permeability [5]. Methicillin and oxacillin resistance arises from the production of penicillin-binding protein 2a (PBP2a), which is encoded by *mec*A and has a low affinity to methicillin and other β-lactams [9]. Furthermore, methicillin resistant *S*. *aureus* (MRSA) can arise from chromosomal mutations that alter the drug binding sites, and from horizontal gene transfer of the mobile genetic element; staphylococcal cassette chromosome elements (*SCCmec*) [10, 11]. MRSA is often resistant to many clinically used antibiotics, such as β-lactams, fluoroquinolones, aminoglycosides, macrolides and lincosamides in Egypt and worldwide [7, 8, 12].

One of the major antimicrobial resistance mechanisms is the efflux pumps [4]. They are membrane proteins that bind to substrates (often are antibiotics), and actively catalyze their translocation in an outward direction leading to a reduction in their intracellular concentrations and thus decrease the therapy efficacy [13, 14]. Therefore, efflux systems can confer a multiple antimicrobial drugs resistance, causing serious public health concerns worldwide [15].

The efflux systems are classified into two categories based on the mechanism by which they derive energy. The primary efflux pumps obtain energy by ATP hydrolysis, whereas the secondary efflux pumps derive energy from chemical gradients formed by protons or ions [16]. Secondary active transporters are highly substrate specific and their recognition sites are often antimicrobial drugs targets [17].

Five major efflux pump families have been found in prokaryotes: ATP binding cassette (ABC) family, small multidrug resistance (SMR) family, multidrug and toxin extrusion (MATE) family, major facilitator superfamily (MFS) and resistance nodulation cell division (RND) family. The ABC family is primary active transporters, while, MFS, SMR, MATE and RND families are secondary active transporters [16]. Bacterial efflux systems can be specific, extruding only one antibiotic class or MDR, extruding several antibiotic classes [18].

The genome of *S*. *aureus* contains more than 30 putative efflux pumps, which belong to MFS, SMR, MATE and RND families [19, 20]. Efflux pumps are encoded by genes that may be present in the chromosome or carried on plasmids [21]. The gene, *msr*A (involved in efflux of erythromycin, azithromycin and clindamycin), and *tet*K, *fex*A and *nor*A genes which involved in the efflux of doxycycline, chloramphenicol and ciprofloxacin, respectively, have been identified in *S*. *aureus* [22].

The continuous emergence of antibiotic-resistant bacteria urges the searching for alternative therapeutic options. A combination therapy of efflux pump inhibitors (EPIs) and antibiotics can be a promising option for the efflux-mediated bacterial resistance [23]. EPIs are compounds having the ability to inhibit the efflux pumps and thus increase the intracellular antibiotic concentration. For any compound to be used as an EPI, it must be selective, not target any eukaryotic efflux pumps, has ideal pharmacological properties, such as the non-toxicity and the high therapeutic effect [24]. Many EPIs have been discovered, but, none of them was clinically approved because of their low potency, narrow activity spectrum, inappropriate pharmacokinetics or high toxicity [11, 25].

Screening the U.S. Food and Drug Administration (FDA)-approved drugs for having EPI activity is safer than the discovery of de novo EPIs as their pharmacokinetics and side effects are well known [26]. Domperidone is an FDA-approved antiemetic drug that had been found to have new uses. It enhanced the activity of ciprofloxacin and levofloxacin in the MDR *E*. *coli* [27]. Furthermore, when combined with omeprazole, it attenuated *Helicobacter pylori* infection [28].

Glyceryl trinitrate (GTN) is an FDA-approved hypotensive drug that has antimicrobial and wound healing activities [29] and approved for the topical treatment of anal fissures [30]. In addition, GTN was found to be able to eradicate the biofilm formation in *S*. *aureus*, *S*. *epidermidis*, and *Pseudomonas aeruginosa* [31, 32]. Moreover, it has an antimicrobial activity against *Candida albicans* [29]. Metformin is one of the most commonly used FDA-approved hypoglycemic drugs for treatment of diabetes mellitus type II [33]. Also, it is able to block the transcription of MDR1 gene that encodes the mammalian P-glycoproteins (P-gp) formation in cancer cells and thus inhibits its expression [34]. Therefore, it inhibits the breast cancer growth and is used in its treatment [33]. Diclofenac sodium is an FDA-approved non-steroidal anti-inflammatory drug (NSAID). It was used as anti-virulence agent in combination with antibiotics for the treatment of *S*. *aureus* osteomyelitis [35]. Verapamil is an ion channel blocker that is used as EPI and treat the hypertension [6]. Also, it is an inhibitor of the mammalian MDR that has a substrate overlap between mammalian P-gp and the bacterial efflux pumps and thus can inhibit the bacterial efflux pumps [36]. Verapamil has been found to enhance ofloxacin and bedaquiline activities by inhibiting MATE pumps activities [37].

This study aimed to investigate efflux pumps in *S*. *aureus* as a mechanism of antibiotic resistance and evaluate the ability of domperidone, diclofenac, GTN, metformin and verapamil as potential EPIs to combat the antibiotic resistance in *S*. *aureus*.

In the current study, the antibiotic susceptibility was determined by disc diffusion method. The efflux was detected phenotypically by EtBr-Cartwheel method and genotypically by PCR to detect four efflux genes; *tet*K, *nor*A, *fex*A and *msr*A. The five compounds were tested for having EPI activities by Cart-Wheel method, efflux assay, the decrease in the antibiotics MICs and RT-PCR that detected the decrease in the expression levels of efflux genes.

## Materials and methods

### 1. Media and chemicals

Ethidium bromide (EtBr), verapamil and the powders of ciprofloxacin, doxycycline, chloramphenicol, azithromycin, erythromycin and clindamycin were obtained from Sigma-Aldrich, St. Louis, USA. Acridine orange (AO; N, N, N’, N’-tetramethylacridine-3, 6-diamine) was purchased from Avonchem, Cheshire, UK. Tryptone soya agar (TSA), tryptone soya broth (TSB) and antibiotics discs were purchased from Oxoid, Hampshire, UK. Müeller-Hinton agar (MHA) and Müeller-Hinton broth (MHB) were obtained from Bioworld, Dublin, USA. The standard strain, *S*. *aureus* ATCC 25923 was from Microbiological Resources Centre, Cairo Mircen Centre, Egypt. PCR-quality water and SuperScript^™^ IV One-Step RT-PCR kit were the products of Thermo Fisher Scientific Inc., USA. MyTaq^™^ red Master Mix was purchased from Bioline, USA. The 100 bp DNA Ladder Marker was from Enzynomics, South Korea. The primers used in this study were obtained from Willowfort, Birmingham, England. Molecular grade agarose was from Bioline, UK. The RNA Purification Kit, Direct-zol^™^ RNA Miniprep Plus was the product of Zymo Research Corp., USA. Domperidone and metformin (1,1-dimethylbiguanide hydrochloride) were the products of EIPICO, 10th of Ramadan City, Egypt. Glyceryl trinitrate "Nitronal^®^" was the product of Sunny Medical, Egypt, while, diclofenac sodium "Voltaren^®^" was produced by Novartis, Switzerland.

### 2. Bacterial isolates

A total of 893 clinical specimens were collected from patients with diabetic foot infections, burn infections, wound infections, urinary tract infections, respiratory tract infections and blood infections who admitted to Zagazig University Hospitals and El-Ahrar educational hospital in Zagazig and Central Hehia Hospital in Hehia, Sharkia, Egypt. These specimens were obtained from the clinical laboratories of the hospitals (collected for routine lab diagnosis) and weren’t directly taken from patients (secondary use). Therefore, there wasn’t any direct or indirect contact with any patient and thus no informed consent or ethical approval was required. All experiments and study protocols complied with relevant guidelines, regulations, and standards of the ethical committee of Faculty of Pharmacy, Zagazig University. The collected (893) clinical specimens yielded 209 clinical *S*. *aureus* isolates.

### 3. Antimicrobial susceptibility testing

Antibiotic susceptibility of isolates was determined by disc diffusion method according to the Clinical and Laboratory Standard Institute CLSI [38] guidelines. The tested antibiotics were: penicillin, oxacillin, cefoxitin, ampicillin/sulbactam, amoxicillin/clavulanic acid, cefuroxime, cefoperazone, cefepime, imipenem, erythromycin, azithromycin, clindamycin, ciprofloxacin, norfloxacin, doxycycline, chloramphenicol, gentamicin, amikacin, sulfamethoxazole/trimethoprim, rifampin, nitrofurantoin, linezolid and novobiocin.

Briefly, an overnight culture of each isolate in MHB were made. Then, the turbidity of culture was adjusted with sterile saline to make them equivalent to 0.5 McFarland standard. Sterile cotton swabs were dipped in the bacterial suspensions, pressed to remove the excess suspension, then streaked over MHA plates surfaces in three directions. Antibiotic discs were placed on the surface of dried inoculated plates so that each plate contained 5 antibiotic discs. Plates were inverted and incubated at 37°C for 18 h. Then, diameters of inhibition zones around discs were measured and interpreted as sensitive (S), intermediate (I) or resistant (R) according to the CLSI [39] guidelines. *S*. *aureus* ATCC 25923 was used as a reference strain. Isolates that were found resistant to at least three different antimicrobial classes were considered MDR [40].

### 4. Confirmation of *mec*A-mediated resistance and detection of vancomycin resistance

The susceptibility of the isolates to oxacillin, cefoxitin or vancomycin was determined by agar dilution method according to CLSI [38, 39]. MHA plates containing 4 μg/mL of cefoxitin, 6 μg/mL oxacillin and 4% NaCl, or 6 μg/mL vancomycin were prepared. Isolates were cultured in TSB at 37°C for overnight, then, their turbidities were adjusted with a sterile saline to be equivalent to 0.5 McFarland. Then, MHA plates were inoculated with 1 μL (in case of oxacillin and cefoxitin) or 2 μL (in case of vancomycin) of bacterial suspensions. Plates were incubated at 33–35°C for 16–20 h, 35°C for 24 h and 37°C for 24 h in case of cefoxitin, oxacillin and vancomycin plates, respectively. Any growth was examined carefully under transmitted light and the presence of more than one colony or the presence of light growth film indicated methicillin resistance, oxacillin resistance or vancomycin resistance.

### 5. Genotypic detection of efflux resistance genes by polymerase chain reaction (PCR)

Depending on the antibiotic resistance profile of the isolates, 72 isolates were selected for further detection of efflux. The phenotypic detection of efflux was confirmed genotypically by PCR, where the presence of the genes encoding the efflux pumps was investigated. The targeted genes were *msr*A which implicated in the efflux of erythromycin, azithromycin and clindamycin and *tet*K, *fex*A, and *nor*A that implicated in efflux of doxycycline, chloramphenicol and ciprofloxacin, respectively. Sequences of primers used in the current study are shown in Table 1.

**Table 1:** Sequences of primers used in this study and amplicon sizes.

Gene	Primer	Primer sequence (5’-3’)	Amplicon size (bp)	Reference
*tet*(K)	*tet*K F	TATTTTGGCTTTGTATTCTTTCAT	1159	[41]
	*tet*K R	GCTATACCTGTTCCCTCTGATAA		
*nor*(A)	*nor*A F	TTCACCAAGCCATCAAAAAG	620	[42]
	*nor*A R	CTTGCCTTTCTCCAGCAATA		
*fex*(A)	*fex*A F	GTACTTGTAGGTGCAATTACGGCTGA	1272	[43]
	*fex*A R	CGCATCTGAGTAGGACATAGCGTC		
*msr*(A)	*msr*A F	TCCAATCATTGCACAAAATC	163	[44]
	*msr*A R	AATTCCCTCTATTTGGTGGT		
16S-rRNA	16S rRNA F	AAACTCAAAKGAATTGACGG	136	[45]
	16S rRNA R	CTCACRRCACGAGCTGAC		

The genomic DNA (gDNA) was extracted by suspending 2 colonies in 20 μL of PCR-quality water, vortexed for 10 s and heated at 95°C for 10 min in Biometra T-personal thermocycler (Goettingen, Germany). The lysate was centrifuged at 13000 rpm for 30 s and the resulting supernatant containing DNA was collected, diluted with distilled water at a 1:3 ratio and subjected to PCR in the thermocycler [46]. Amplification mixture was prepared in a final volume of 25 μL and consists of: 12.5 μL of MyTaq^™^ red Master Mix, 1.5 μL of each of forward and reverse primers, 7.5 μL of nuclease free water and 2 μL of gDNA. A reaction containing all PCR components except DNA was used as negative control. Conditions for amplification of genes are listed in Table 2. After amplification, PCR products and the DNA ladder were resolved using 1% agarose gel, stained with 2 μL of EtBr solution (5 μg/mL), separated according to its molecular size by electrophoresis and visualized on UV transilluminator (Hoefer, USA) [47].

**Table 2:** Amplification reaction cycles of PCR.

Gene	Initial denaturation	Number of cycles	Denaturation	Annealing	Extension at 72°C for	Final extension at 72°C for
*fex*A	94°C / 2 min.	35	94°C / 1 min.	56°C / 2 min.	3 min.	7 min.
*tet*K	95°C / 1 min.	35	95°C / 60 sec.	50°C / 1 min.	60 sec.	5 min.
*nor*A	94°C / 4 min.	35	94°C / 30 sec.	45°C / 30 sec.	60 sec.	5 min.
*msr*A	96°C / 3 min.	35	96°C / 45 sec.	57°C / 45 sec.	45 sec.	7 min.

### 6. Qualitative detection of efflux by ethidium bromide Cart-Wheel method

Efflux pump activity was detected in the selected 72 isolates according to EtBr Cart-Wheel method of Martins *et al*. [48, 49]. Briefly, five sets of TSA plates containing different EtBr concentrations ranging from 0.5 to 4 mg/L were prepared. Each plate was divided into 6 sectors by radial lines. Standardized suspensions of isolates (0.5 McFarland) were prepared from overnight cultures in TSB and streaked over plates starting from the center till the plate edges. Each plate included the standard strain, *S*. *aureus* ATCC 25923 that served as a negative efflux control [42]. Plates were incubated at 37°C for 16 h and after incubation they were examined under UV transilluminator. Minimum concentration of EtBr that produces a clear fluorescence was recorded for each isolate. The higher the EtBr concentration that produced a clear fluorescence, the greater the efflux capacity of the isolate.

### 7. Detection of the efflux pump inhibitory activities of the tested drugs

To investigate the efflux further and detect the potential EPI activities of the tested drugs, we selected 26 isolates. In EtBr Cart-Wheel assay and PCR, these isolates showed an efflux activity and harbored at least one of the four efflux genes. For testing drugs, the following five FDA-approved drugs were used: diclofenac sodium, GTN, domperidone, metformin and verapamil (used as a control EPI).

#### 7.1. Determination of minimum inhibitory concentrations of the tested compounds

Minimum inhibitory concentrations (MICs) of GTN and diclofenac sodium were determined by broth microdilution method, while, MICs of domperidone, metformin and verapamil were determined by agar dilution method according to CLSI [38]. In both methods, 4 well-isolated colonies of each isolate were transferred from an overnight MHA plate culture to 5 mL saline and the turbidity was adjusted to 0.5 McFarland standard. In broth microdilution method, the bacterial suspensions were diluted 1:100 in MHB so that the final concentration of bacteria was about 5 × 10^5^ CFU/mL. While, in agar dilution method, the adjusted bacterial suspensions were diluted 1:10 in a sterile saline.

In broth microdilution method, double strength of two-fold serial dilutions of each tested compound were prepared directly in 96 wells microtiter plates in a final volume of 50 μL per well. One well was left without inhibitor and served as positive growth control. Each well was inoculated with 50 μL of inoculum and incubated at 37° for 18 to 20 h. The microtiter plates were examined for growth.

In agar dilution method, fresh stock solutions of the tested compounds were prepared using sterile water as a solvent. Two-fold serial dilutions of the tested compounds were prepared and added to sterile molten MHA at 45–50°C and each plate contained different concentration of the tested compound. One μL of the bacterial suspension was delivered to the surface of MHA plates containing the tested compound dilutions and control plates that containing drug-free agar, so that the final inoculum is approximately 10^4^ CFU/spot. Sixteen isolates were applied to each plate. The plates were inverted and incubated at 37°C for 18–24 h. In both methods, MIC was considered as the lowest concentration at which there was no visible growth of the organism.

#### 7.2. Qualitative estimation of efflux inhibitory effect of tested drugs by Cart-Wheel method using AO

The abilities of the isolates to efflux AO were detected according to Martins *et al*. [48] in which the assessment of efflux inhibition was performed in the presence of each of the five tested drugs.

In the presence of the tested drug, the lower the concentration of AO that produced a high fluorescence, the greater the efflux inhibition activity of the tested drug. Six sets of TSA plates containing different AO concentrations ranging from 0.5 to 20 mg/L were prepared. One set of plates contained AO only served as negative control (represent the original efflux capacity of the isolates). The second set of plates contained AO and 1/8 MIC of verapamil which served as positive control. The remaining 4 sets of plates contained AO and 1/8 MIC of one of the four tested drugs (diclofenac sodium, GTN, domperidone and metformin). To avoid any possibility of inhibiting the growth of isolates that could affect the efflux inhibition, the tested drugs were used at their sub-MICs (1/8 MIC) that showed no effects on the isolates’ growth. Plates were prepared in the same way as the EtBr Cart-Wheel method. The effects of the tested drugs on the efflux activity of each isolate were examined and the lowest concentration of AO that showed a fluorescence of the bacterial streak was recorded.

#### 7.3. Quantitative determination of efflux inhibitory effect of tested drugs by semi-automated fluorometric efflux assay

*7*.*3*.*1*. *Determination of AO-MIC and the effect of the sub-MIC of tested drugs on AO-MIC*. According to Paixão *et al*. [50], the MIC of AO alone and in the presence of 1/8 MIC of one of the 5 tested compounds were determined either by broth micro-dilution method or agar dilution method according to CLSI [38] guidelines.

*7*.*3*.*2*. *Effect of tested compounds on AO accumulation*. The accumulation of AO was carried out according to Paixão *et al*. [50]. This test was performed to determine the concentration of AO that causes its maximum accumulation. Therefore, the conditions that minimize the efflux of AO from the cells (25°C and the absence of glucose) were employed. Isolates were grown in 10 mL of TSB until they reached a mid-log phase (the optical density at 600 nm (OD_600_) equals 0.6 that match 6 McFarland standard). The bacteria were then centrifuged at 14000 rpm for 3 min. The cell pellets were washed twice with the same volume of phosphate buffered saline (PBS) and the OD_600_ of the cellular suspensions adjusted to 0.3 (match that of 1 McFarland standard).

The accumulation assays were performed in 96 well microtiter plate with a final volume of 100 μL where 50 μL of each washed cell suspension was added to 50 μL of varying concentrations of AO in the absence of glucose at 25°C. During the preparation, plates were kept in ice and all the solutions were cold. Then, the fluorescence was measured using ELISA reader (Biotek, USA) at 25°C at 0 time and after 1 h and at the excitation (502 nm) and emission (525 nm) wavelengths for AO to monitor AO accumulation on a real-time basis.

After determination of AO concentration that causes its maximum accumulation (30 μg/mL), the effect of each of the tested compounds on AO accumulation was determined (to determine the appropriate concentration of EPI to be used in the efflux assay and causes the maximum AO accumulation) where the conditions that optimize the efflux (37°C and the presence of glucose) were employed [50]. A volume of 50 μL of washed cell suspension was added to 50 μL PBS solutions containing 30 μg/mL of AO, 0.4% glucose and different concentrations of one of the tested EPIs (beginning from their 1/4 MIC to less). Then, the fluorescence was measured at 37°C at 0 time and after 1 h and at 502 nm and 525 nm wavelengths.

*7*.*3*.*3*. *Assay of efflux activity in the presence of tested compounds*. According to Paixão *et al*. [50], the cells were exposed to conditions that promoted the maximum accumulation of AO (by using the appropriate concentrations of EPI and AO determined in the previous step, a temperature of 25°C and the absence of glucose). The isolates were grown in 5 mL of TSB and incubated at 37°C for 18 h. After incubation, the isolates were centrifuged at 14000 rpm for 5 min. The supernatants were discarded and the cell pellets were obtained. Then, the maximum AO accumulation conditions were promoted by adding the cell pellets to 30 μg/mL of AO and one of the tested compounds at the concentrations that caused the maximum AO accumulation. Then, the tubes (containing the cell pellet, AO and EPI) were incubated at 25°C at 200 rpm for 1 h to allow loading the cells with AO (by minimizing the efflux and causing the maximum AO accumulation).

After incubation, the tubes were centrifuged at 13000 rpm for 5 min and the supernatants were discarded. Then, cell pellets were washed with cold PBS to minimize the efflux, by adding 2 mL of PBS, inverting the tubes several times and centrifugation at 13000 rpm for 5 min. Supernatants were discarded and the pellet was suspended in 1 mL of cold PBS.

Then 50 μL of each washed isolate cell suspension was added in the 96 well plate to each of the following: i) 50 μL of PBS solution containing 0.4% glucose (positive efflux control); ii) 50 μL of PBS solution containing 1/8 MIC of tested compounds (negative efflux control); iii) 50 μL of PBS solution only. During the preparation, plates were kept in ice and all the solutions were cold. Then, the fluorescence was measured at 37°C at 0 time and after 1 h and at 502 nm and 525 nm wavelengths. The assay was performed in triplicate.

The efflux of AO was monitored by the decrease in the fluorescence of the cell suspension. It was presented in terms of relative fluorescence (RF), which is obtained by comparing the fluorescence of the treated cells (in the absence of glucose) and the fluorescence of the untreated cells (in the presence of glucose) with the control cells that have the maximum fluorescence (in the absence of glucose and the presence of EPI).

In addition, the difference between the RF of treated cells and the RF of untreated cells is the relative final fluorescence (RFF). As the RFF increases, the EPI activity of the tested compound increases. Therefore, the tested compound which has the highest RFF, will supposed to have the most potent EPI activity.


$${\text{RF}}_{\text{treated}}=\text{fluorescence}\;\text{of}\;\text{the}\;\text{treated}\;{\text{cells}}_{\text{without}\;\text{glucose}}/\text{maximum}\;\text{fluorescence}\;\text{of}\;\text{control}\;\text{cells}$$

$${\text{RF}}_{\text{untreated}}=\text{fluorescence}\;\text{of}\;\text{the}\;\text{untreated}\;{\text{cells}}_{\text{in}\;\text{the}\;\text{presence}\;\text{of}\;\text{glucose}}/\text{maximum}\;\text{fluorescence}\;\text{of}\;\text{control}\;\text{cells}.$$

$$\text{RFF}={\text{RF}}_{\text{treated}}{-\text{RF}}_{\text{untreated}}$$

#### 7.4. Investigating the effect of sub-MICs of tested compounds on antimicrobials MICs

The MICs of ciprofloxacin, doxycycline, chloramphenicol, azithromycin, erythromycin and clindamycin alone and in the presence of 1/8 MICs of the tested compounds were determined by broth microdilution method according to CLSI [38] as mentioned above. In case of antibiotics whose MICs were above 1024 μg/mL and thus their stock solutions became turbid at high concentrations, their MICs were determined by agar dilution method according to CLSI [38].

#### 7.5. RNA extraction and the measurement of relative expression of efflux genes by qRT-PCR

Five isolates that showed significant reduction in their efflux in presence of the tested drugs were selected to confirm the effects of domperidone, GTN and diclofenac sodium on the expression levels of efflux genes (*nor*A, *fex*A and *tet*K) by quantitative real time-PCR (qRT-PCR).

According to Kong *et al*. [51], the isolates were cultured in TSB in the absence (control) and presence of 1/8 MICs of domperidone (750, 875 and 1250 μg/mL), GTN (125 μg/mL) and diclofenac sodium (48.8 μg/mL) at 37°C for 16–18 h with simple agitation. Then, the tubes were centrifuged at 6000 rpm for 5 min and the supernatants were discarded to obtain the pellets. Then, RNA was extracted from each pellet using Direct-zol^™^ RNA Miniprep Plus (RNA Purification Kit).

Reverse transcription followed by the qRT-PCR of efflux genes were performed following the protocol described in SuperScript^™^ IV One-Step RT-PCR kit. The qRT-PCR analysis was carried out by RT-PCR thermal cycler (Step One Applied Biosystem, USA) using the primers mentioned before in Table 1. Each gene relative expression value was normalized against house-keeping gene (16S-rRNA). The relative gene expression of treated isolates was compared to their expression in the untreated ones according to the 2^−ΔΔCT^ method [52].

### 8. Statistical analysis

The significance of correlations between the susceptibilities of isolates to ciprofloxacin, doxycycline, chloramphenicol, azithromycin, erythromycin and clindamycin and the prevalence of efflux genes was analyzed by Chi-square test analysis using IBM SPSS Statistics for Windows software version 25.0 (IBM Corp., USA). P value of *<0*.*05* was considered statistically significant. In addition, the significance of the inhibitory activities of domperidone, GTN and diclofenac sodium on the expression of efflux genes (*tet*K, *nor*A and *fex*A) were analyzed using GraphPad Prism 9 software where One Way ANOVA analysis followed by Dunnett’s Multiple Comparison tests were performed. P values of <*0*.*05* and <*0*.*001* were considered statistically significant.

## Results

### 1. Identification of *Staphylococcus aureus* isolates

Two hundred and nine clinical *S*. *aureus* isolates were included in this study. The isolates were identified microscopically as Gram-positive cocci and biochemically by producing catalase, coagulase and urease, producing a golden yellow pigment and β-hemolysis, fermenting mannitol and sucrose but not fermenting arabinose, liquefying the gelatin and being novobiocin sensitive [53].

### 2. Antimicrobial susceptibility of isolates

The diameter of each inhibition zone around the tested antibiotics was measured and showed in S1 Table. Then, the results were interpreted according to the zone diameter breakpoints of CLSI [39] (S2 Table) into susceptible (S), intermediate (I) and resistant (R). The number and the percentages of susceptible, intermediate and resistant isolates of tested antibiotics are shown in S3 Table.

**Fig 1:**
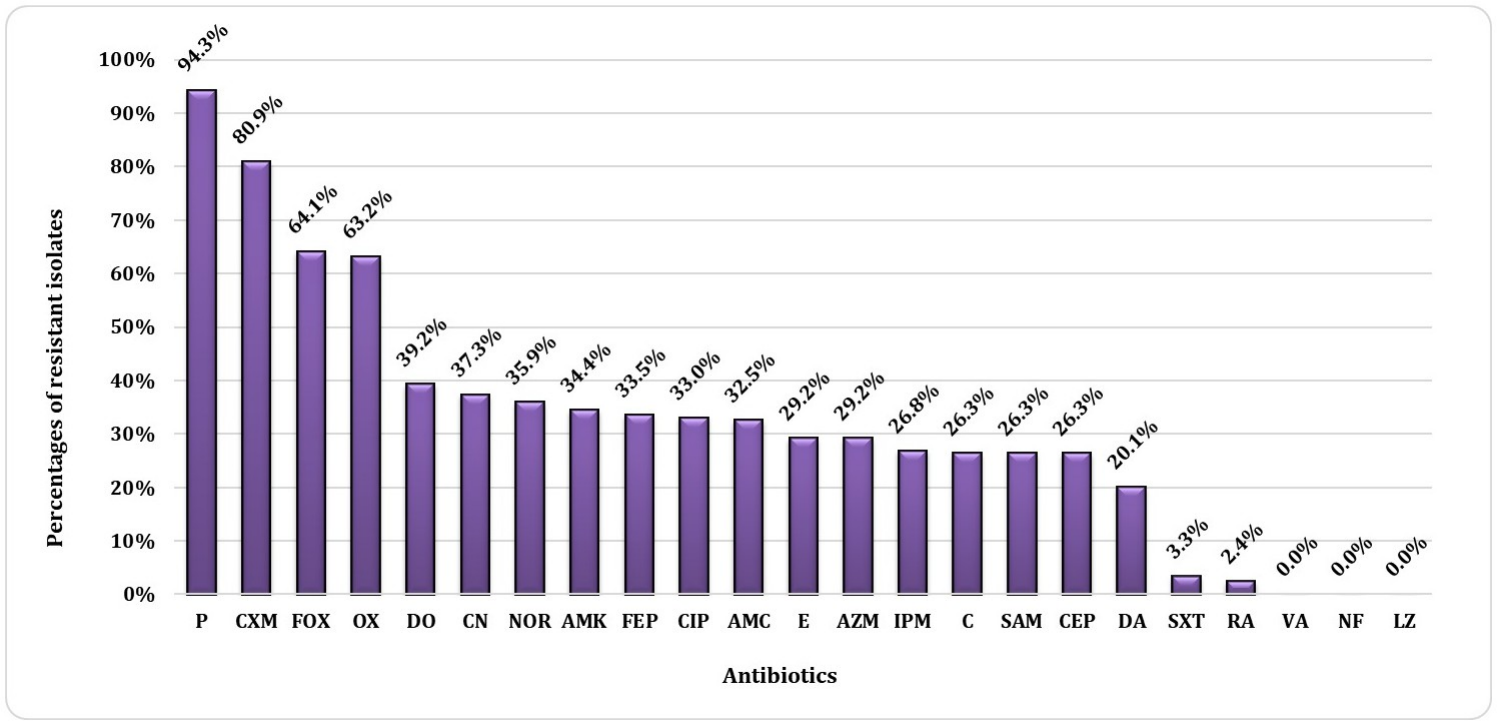
Antibiotic resistance profile of *S*. *aureus* isolates. The percentages shown in the figure represent the percentages of the resistant isolates to each tested antibiotic. P, penicillin G; CXM, ceforuxime; FOX, cefoxitin; OX, oxacillin; DO, doxycycline; CN, gentamicin; NOR, norfloxacin; AMK, amikacin; FEP, cefepime; CIP, ciprofloxacin; AMC, amoxicillin / clavulanic acid; E, erythromycin; AZM, azithromycin; IMP, imipenem; C, chloramphenicol; SAM, ampicillin / sulbactam; CFP, cefoperazone; DA, clindamycin; SXT, sulphamethoxazole / trimethoprim; RA, rifampin; VA, vancomycin; NF, nitrofurantoin and LZ, linezolid.

From the total 209 isolates, 94.3% were resistant to penicillin G and 80.9% were resistant to cefuroxime. In addition, 64.1% were resistant to cefoxitin and 63.2% were resistant to oxacillin. Only 3.3% and 2.4% of the total isolates were resistant to sulphamethoxazole/trimethoprim and rifampin, respectively. On the other hand, no resistance was found to linezolid, nitrofurantoin or vancomycin (all isolates were sensitive). The percentages of resistance against the tested antibiotics are shown in Fig 1.

Isolates that were found resistant to oxacillin and cefoxitin, were confirmed by agar dilution method and considered MRSA (64.1%). From the total tested (209) isolates, 78 (37.3%) isolates were found MDR.

### 3. The prevalence of efflux pump genes among the isolates

Based on the antibiotic sensitivity results, 72 isolates were selected and subjected to PCR. These selected isolates consisted of: 60 MDR MRSA, 6 non MDR MRSA and 6 MDR MSSA isolates. The profile of the 72 selected isolates is shown in S4 Table.

**Fig 2:**
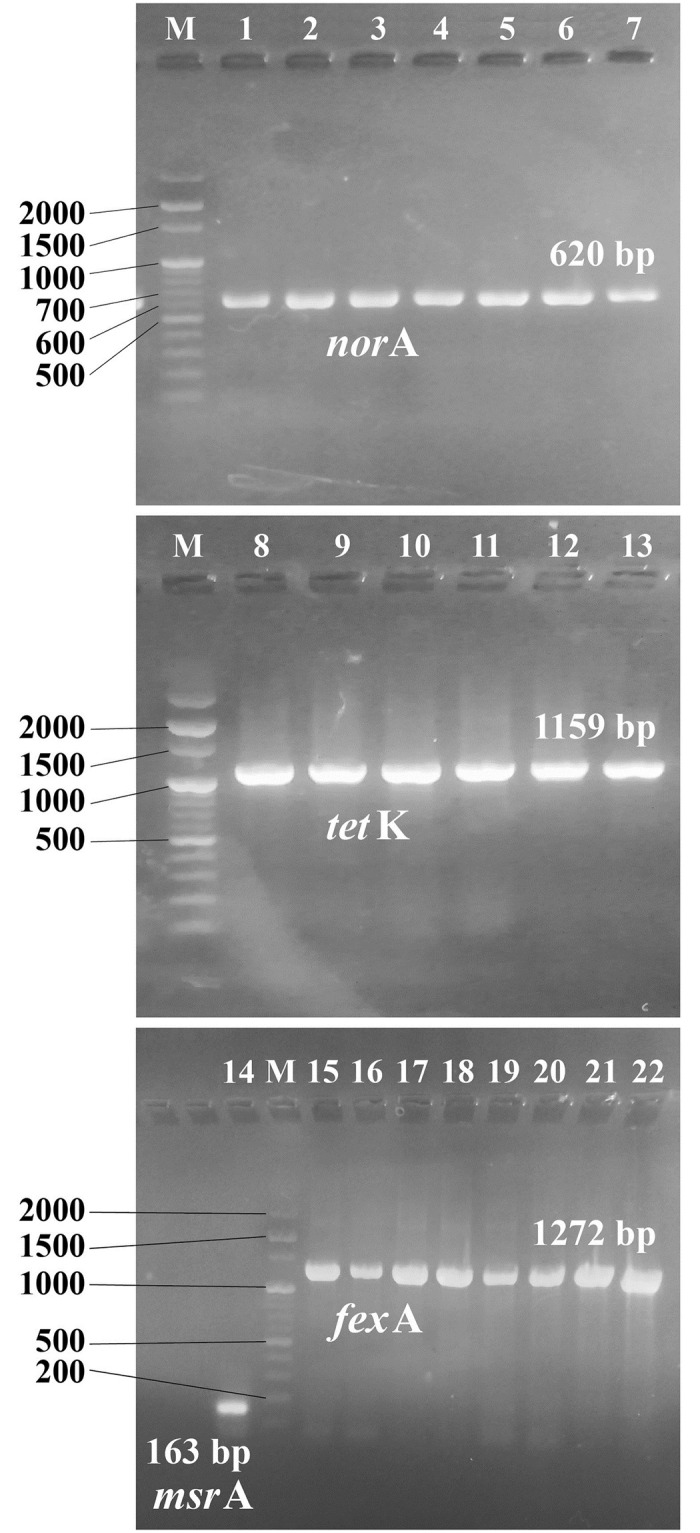
Electrophoretic analysis of PCR products of detected efflux genes. M; DNA ladder marker (3000 bp), Lanes from 1 to 7; *nor*A gene (620 bp), Lanes from 8 to13; *tet*K gene (1159 bp), Lane 14; *msr*A gene (163 bp) and Lanes from 15 to 22; *fex*A gene (1272 bp).

PCR amplification of the tested genes revealed that the prevalence of *nor*A, *tet*K, *fex*A and *msr*A genes were 91.7%, 77.8%, 27.8% and 6.9%, respectively. Some examples are shown in Fig 2.

The possible correlations between the susceptibilities of isolates to the tested antibiotics and the presence of the efflux genes were investigated by testing the significance of the difference between them using Chi-square test. The statistical analysis revealed that no significant difference was found between the antibiotic resistance and the efflux genes (*P = 1*.*0*). These results indicate that the efflux gene content cannot explain the antibiotic resistance, and other resistance mechanisms (e.g., target site modification and enzymatic inactivation of antibiotics) could contribute to the resistance. In addition, 98.5% of ciprofloxacin resistant isolates harbored *nor*A gene and 92.9% of doxycycline resistant isolates harbored *tet*K gene. While, 47.9% of chloramphenicol resistant isolates harbored *fex*A gene. On the other hand, only 11.1% of erythromycin resistant isolates, 9.1% of azithromycin resistant isolates and none of clindamycin resistant isolates harbored *msr*A gene. The number of resistant isolates that harbored the efflux genes from which the statistical analysis was made is provided in S5 Table.

### 4. Qualitative detection of efflux by ethidium bromide Cart-Wheel method

**Fig 3:**
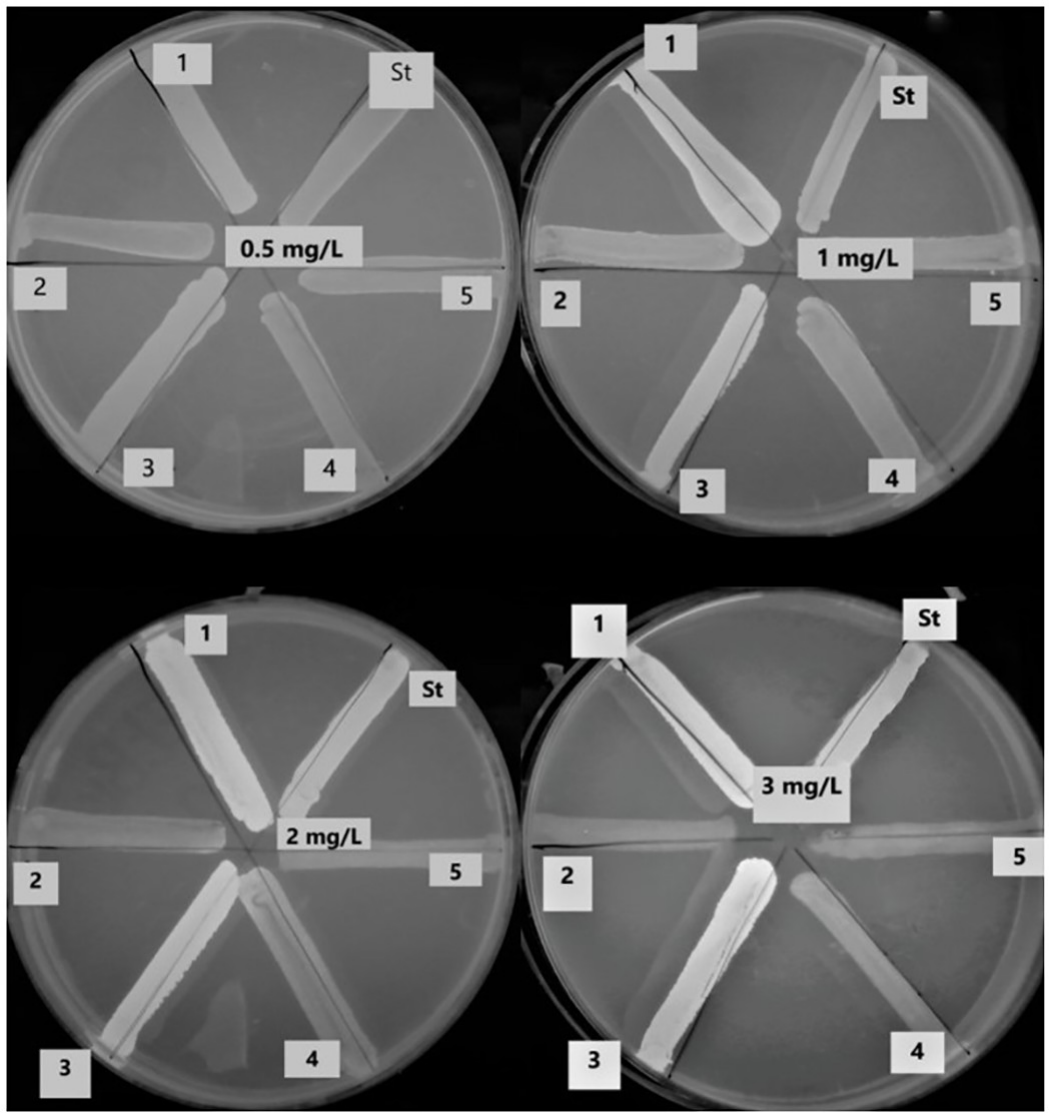
Representative example of different efflux capacities of 5 tested isolates and 1 standard isolate (*S*. *aureus* ATCC 25923) at different EtBr concentrations (from 0.5 to 3 mg/L) under UV transilluminator showing positive fluorescence (negative efflux; Isolates 1, 3 and St), negative fluorescence (positive efflux; Isolates 2, 4 and 5).

The selected 72 isolates were subjected to the qualitative assessment of efflux by EtBr Cart-Wheel method, and the minimum EtBr concentration producing a fluorescence (MC-EtBr) was determined. The isolates that had MC-EtBr higher than 2 μg/mL were considered to have positive efflux activity, while, the isolates that had MC-EtBr equal to 2 μg/mL were considered to have intermediate efflux activity. In addition, the isolates that had MC-EtBr at 1 μg/mL or less were considered negative. Out of the 72 isolates, 18 isolates (25.0%) were EtBrCW-positive, 29 (40.3%) were EtBrCW-intermediate and 25 isolates (34.7%) were EtBrCW-negative. The standard strain, *S*. *aureus* ATCC 25923, was EtBrCW-negative. The results are shown in S6 Table. A representative example of different efflux capacities of 6 tested isolates at different EtBr concentration (from 0.5 to 3 mg/L) is shown in Fig 3.

### 5. Detection of efflux pump inhibitory activities of tested drugs

From the 72 isolates, screened by PCR and EtBr Cart-Wheel method for the presence of efflux activity, 26 isolates were selected for further investigation of efflux and detecting the EPI activities of the tested drugs. These selected (26) isolates had efflux activities and contained one or more of the tested efflux genes (S7 Table). They consisted of 16 EtBrCW-positive isolates and 10 EtBrCW-intermediate isolates. In addition, *S*. *aureus* ATCC 25923 was taken as a negative efflux control (EtBrCW-negative).

#### 5.1. The minimum inhibitory concentration (MIC) of tested compounds

The MICs of the 5 tested compounds were determined. The MIC values of verapamil were 64, 512, 1200 and 1500 μg/mL, of domperidone were 6000, 7000 and 10000 μg/mL, of GTN was 1000 μg/mL, of diclofenac sodium was 390.6 μg/mL and of metformin was 10000 μg/mL.

#### 5.2. Qualitative detection of tested compounds effect on the efflux of isolates

**Fig 4:**
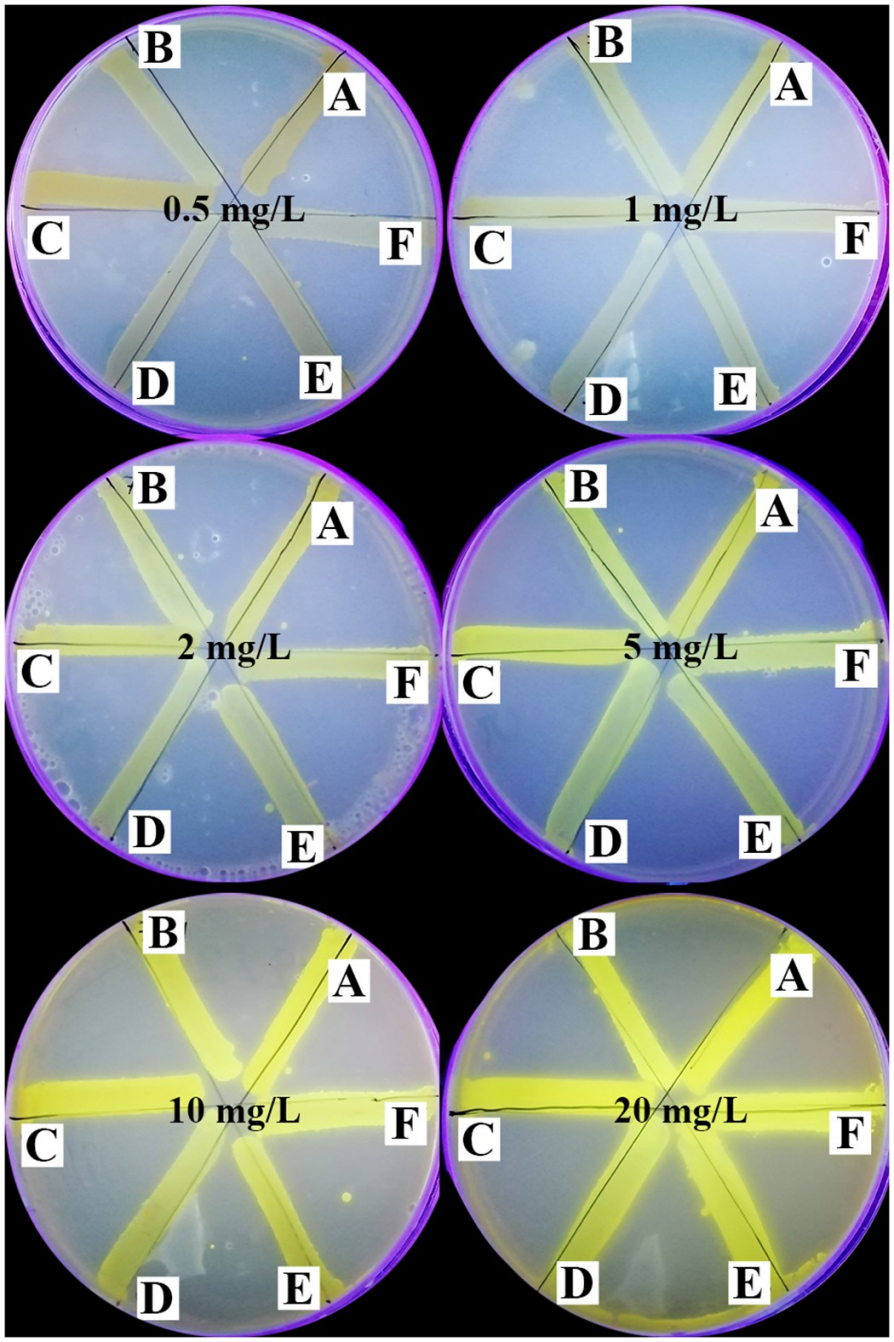
A representative example of different efflux capacities of 6 tested isolates at different AO concentrations (from 0.5 to 20 mg/L) under UV transilluminator showing positive fluorescence (negative efflux; Isolates A and C) and negative fluorescence (positive efflux; Isolates B, D, E and F).

The efflux of AO in the absence of tested compounds was assessed by Cart-Wheel method using AO in the selected 26 isolates. The higher the concentration of AO that produced a clear fluorescence, the greater was the efflux activity of isolates (S8 Table). A representative example of different efflux capacities of 6 tested isolates at different AO concentrations (from 0.5 to 20 mg/L) is shown in Fig 4.

**Fig 5:**
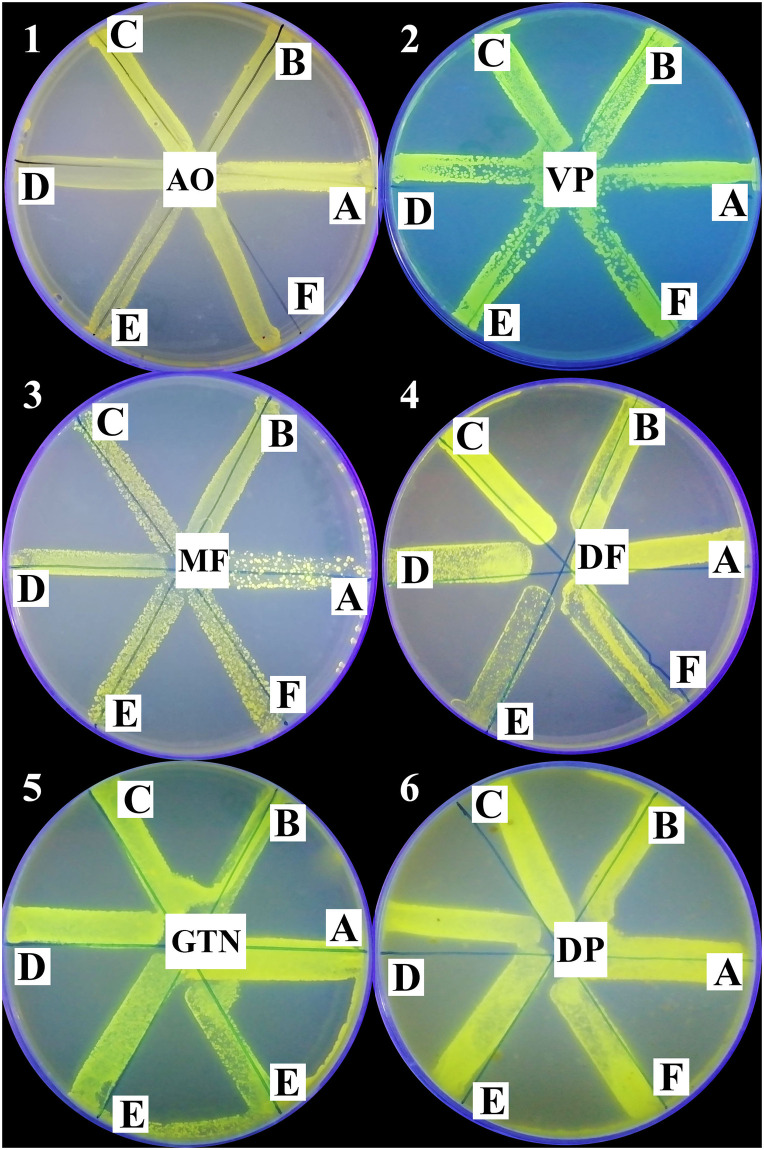
The efflux of 6 representative isolates (A; standard strain and B, C, D, E, and F; 5 tested isolates) in the absence of the tested compounds (acridine orange only; AO, negative control in sector 1) and in the presence of verapamil (VP; positive control in sector 2), metformin (MF in sector 3), diclofenac sodium (DF in sector 4), glyceryl trinitrate (GTN in sector 5) and domperidone (DP in sector 6) at the same AO concentration (10 mg/L).

Furthermore, the efflux of AO in the presence of the 1/8 MIC of each of the 5 tested compounds was assessed by Cart-Wheel method. The lower the concentration of AO that produced a clear fluorescence, the greater was the EPI activity of the tested compound (S9–S13 Tables). The different effects of the tested compounds on the efflux of 6 representative isolates at the same AO concentration (10 mg/mL) are shown in Fig 5.

#### 5.3. Quantitative determination of inhibitory effect of tested compounds on the efflux of isolates

The ability of the selected 26 isolates to efflux AO was assessed by the semi-automated fluorometric efflux assay method. The efflux of AO is presented in terms of RF (relative fluorescence). The lower the RF, the higher efflux pump activity of the isolate and thus a reduction in the fluorescence was observed. The higher the RFF values compared to those of verapamil (control), the higher the efflux inhibitory activity of the tested compound. The median values of RFF of diclofenac sodium, GTN, domperidone, verapamil and metformin were 0.55, 0.43, 0.38, 0.27 and 0.22 arbitrary units, respectively. Therefore, efflux assay revealed that diclofenac sodium, GTN and domperidone had better EPI activities than verapamil, while, metformin had a lower EPI activity. Values of RF and RFF of isolates in the presence of one of the five tested compounds are shown in Tables 3 and 4.

**Table 3:** Fluorometric efflux assay of isolates in the presence of verapamil, domperidone and glyceryl trinitrate.

Isolate code	Verapamil (VP)						Domperidone (D)						Glyceryl trinitrate (GTN)					
	Maximum F (VP, no GL)	Measured F		RF		RFF	Maximum F (D, no GL)	Measured F		RF		RFF	Maximum F (GTN, no GL)	Measured F		RF		RFF
		- GL	+ GL	- GL	+ GL			- GL	+ GL	- GL	+ GL			- GL	+ GL	- GL	+ GL	
**E 189**	37	32	17	0.88	0.50	**0.38**	55	36	18	0.65	0.33	**0.33**	59	43	19	0.73	0.32	**0.41**
**B 866**	32	28	16	0.86	0.46	**0.41**	64	45	15	0.70	0.23	**0.47**	60	50	15	0.83	0.25	**0.58**
**B 3**	36	30	19	0.83	0.53	**0.31**	56	35	14	0.63	0.25	**0.38**	55	47	22	0.85	0.40	**0.45**
**B 50**	37	30	17	0.81	0.46	**0.35**	52	40	21	0.77	0.40	**0.37**	51	43	19	0.84	0.37	**0.47**
**W 898**	32	26	16	0.81	0.50	**0.31**	61	43	15	0.70	0.25	**0.46**	62	48	14	0.77	0.23	**0.55**
**S 417**	31	28	14	0.90	0.45	**0.45**	67	49	19	0.73	0.28	**0.45**	64	48	16	0.75	0.25	**0.50**
**B 868**	30	28	17	0.93	0.57	**0.37**	58	50	27	0.86	0.47	**0.40**	57	41	13	0.72	0.23	**0.49**
**B 774**	31	29	20	0.94	0.65	**0.29**	50	32	14	0.64	0.28	**0.36**	53	41	18	0.77	0.34	**0.43**
**B 786**	36	30	18	0.83	0.50	**0.33**	53	31	13	0.58	0.25	**0.34**	59	50	25	0.85	0.42	**0.42**
**W 914**	38	32	16	0.84	0.42	**0.42**	68	57	18	0.84	0.26	**0.57**	65	54	20	0.83	0.31	**0.52**
**W 628**	46	39	20	0.85	0.43	**0.41**	62	50	17	0.81	0.27	**0.53**	63	55	19	0.87	0.30	**0.57**
**B 97**	33	29	20	0.88	0.61	**0.27**	58	41	16	0.71	0.28	**0.43**	53	40	14	0.75	0.26	**0.49**
**B 776**	27	21	15	0.78	0.56	**0.22**	56	43	23	0.77	0.41	**0.36**	63	55	23	0.87	0.37	**0.51**
**B 864**	40	36	28	0.90	0.70	**0.20**	54	38	14	0.70	0.26	**0.44**	59	48	24	0.81	0.41	**0.41**
**B 84**	39	33	23	0.85	0.59	**0.26**	55	34	14	0.62	0.25	**0.36**	57	50	26	0.88	0.46	**0.42**
**B 21**	31	26	20	0.84	0.65	**0.19**	59	42	17	0.71	0.29	**0.42**	51	44	22	0.86	0.43	**0.43**
**B 783**	32	28	21	0.88	0.66	**0.22**	53	40	23	0.75	0.43	**0.32**	59	48	24	0.81	0.41	**0.41**
**W 823**	29	26	20	0.90	0.69	**0.21**	63	44	21	0.70	0.33	**0.37**	52	46	25	0.88	0.48	**0.40**
**W 871**	33	28	21	0.85	0.64	**0.21**	61	44	18	0.72	0.30	**0.43**	54	43	20	0.80	0.37	**0.43**
**W 820**	35	27	17	0.77	0.49	**0.29**	55	35	16	0.64	0.29	**0.35**	51	40	19	0.78	0.37	**0.41**
**B 48**	34	29	19	0.85	0.56	**0.29**	61	49	21	0.80	0.34	**0.46**	55	44	21	0.80	0.38	**0.42**
**W 873**	29	26	19	0.90	0.66	**0.24**	59	43	20	0.73	0.34	**0.39**	50	45	22	0.90	0.44	**0.46**
**E 444**	29	26	19	0.90	0.66	**0.24**	59	48	24	0.81	0.41	**0.41**	61	49	27	0.80	0.44	**0.36**
**B 31**	31	26	19	0.84	0.61	**0.23**	54	32	19	0.59	0.35	**0.24**	53	41	21	0.77	0.40	**0.38**
**W 446**	25	18	12	0.72	0.48	**0.24**	54	32	16	0.59	0.30	**0.30**	60	48	24	0.80	0.40	**0.40**
**B 26**	20	17	12	0.85	0.60	**0.25**	56	30	12	0.54	0.21	**0.32**	64	52	23	0.81	0.36	**0.45**
**St**.	38	36	33	0.95	0.87	**0.08**	35	25	22	0.71	0.63	**0.09**	35	30	28	0.86	0.80	**0.06**

F, fluorescence; RFF, Relative final fluorescence (arbitrary units); RF, Relative fluorescence (arbitrary units); GL, glucose; + GL, with glucose; − GL, without glucose.

**Table 4:** Fluorometric efflux assay of isolates in the presence of diclofenac sodium and metformin.

Isolate code	Diclofenac sodium (DF)						Metformin (MF)					
	Maximum F (DF, no GL)	Measured F		RF		RFF	Maximum F (MF, no GL)	Measured F		RF		RFF
		- GL	+ GL	- GL	+ GL			- GL	+ GL	- GL	+ GL	
**E 189**	89	70	25	0.79	0.28	**0.51**	31	28	20	0.90	0.65	**0.26**
**B 866**	80	69	20	0.86	0.25	**0.61**	36	34	28	0.94	0.78	**0.17**
**B 3**	88	75	26	0.85	0.30	**0.56**	34	33	25	0.97	0.74	**0.24**
**B 50**	72	60	20	0.83	0.28	**0.56**	30	28	22	0.93	0.73	**0.20**
**W 898**	81	69	19	0.85	0.23	**0.62**	30	29	21	0.97	0.70	**0.27**
**S 417**	84	68	17	0.81	0.20	**0.61**	30	28	19	0.93	0.63	**0.30**
**B 868**	75	65	21	0.87	0.28	**0.59**	27	25	19	0.93	0.70	**0.22**
**B 774**	72	60	22	0.83	0.31	**0.53**	33	29	22	0.88	0.67	**0.21**
**B 786**	81	68	20	0.84	0.25	**0.59**	25	24	19	0.96	0.76	**0.20**
**W 914**	79	64	15	0.81	0.19	**0.62**	44	42	33	0.95	0.75	**0.20**
**W 628**	75	63	16	0.84	0.21	**0.63**	43	40	30	0.93	0.70	**0.23**
**B 97**	68	52	17	0.76	0.25	**0.51**	42	38	30	0.90	0.71	**0.19**
**B 776**	66	53	18	0.80	0.27	**0.53**	32	30	23	0.94	0.72	**0.22**
**B 864**	69	51	16	0.74	0.23	**0.51**	33	30	23	0.91	0.70	**0.21**
**B 84**	73	59	21	0.81	0.29	**0.52**	43	36	29	0.84	0.67	**0.16**
**B 21**	70	53	16	0.76	0.23	**0.53**	25	22	15	0.88	0.60	**0.28**
**B 783**	79	67	21	0.85	0.27	**0.58**	25	20	14	0.80	0.56	**0.24**
**W 823**	81	68	23	0.84	0.28	**0.56**	32	31	24	0.97	0.75	**0.22**
**W 871**	74	61	20	0.82	0.27	**0.55**	27	26	20	0.96	0.74	**0.22**
**W 820**	84	68	20	0.81	0.24	**0.57**	36	34	26	0.94	0.72	**0.22**
**B 48**	78	65	24	0.83	0.31	**0.53**	20	18	13	0.90	0.65	**0.25**
**W 873**	75	59	19	0.79	0.25	**0.53**	28	26	20	0.93	0.71	**0.21**
**E 444**	74	62	20	0.84	0.27	**0.57**	34	31	23	0.91	0.68	**0.24**
**B 31**	71	57	21	0.80	0.30	**0.51**	20	19	14	0.95	0.70	**0.25**
**W 446**	65	55	20	0.85	0.31	**0.54**	32	30	21	0.94	0.66	**0.28**
**B 26**	80	69	27	0.86	0.34	**0.53**	29	26	20	0.90	0.69	**0.21**
**St**.	38	36	35	0.95	0.92	**0.03**	28	26	25	0.93	0.89	**0.04**

F, fluorescence; RFF, Relative final fluorescence (arbitrary units); RF, Relative fluorescence (arbitrary units); GL, glucose; + GL, with glucose; − GL, without glucose.

#### 5.4. MICs of antibiotics and the effect of sub-MICs of tested compounds on antimicrobials MICs

The MICs (μg/mL) of antibiotics in the absence and presence of 1/8 MICs of the tested compounds were determined. The reduction in the MICs of antibiotics to at least the 1/4 of their original values in the presence of tested drugs was considered as indication of their EPI effects as shown in S14–S16 Tables.

The averages of the fold decreases in the MICs of the tested antibiotics in the presence of the tested compounds (shown in Table 5), revealed that the best combinations (had the highest fold decrease in the MIC) are: diclofenac sodium with chloramphenicol, doxycycline or ciprofloxacin, GTN or domperidone with azithromycin and diclofenac sodium or domperidone with erythromycin.

**Table 5:** Averages of the fold decreases in the MICs of tested antibiotics in the presence of the tested compounds.

Antibiotic	Average of fold decrease in the MIC in the presence of				
	DF	D	GTN	VP	MF
Chloramphenicol	158.2	135.8	145.8	26.9	19.8
Doxycycline	121.0	45.1	88.0	14.8	4.5
Ciprofloxacin	52.0	47.8	26.7	12.8	9.5
Azithromycin	32.0	37.3	37.3	5.3	3.0
Erythromycin	29.2	29.2	24.4	18.0	7.0
Clindamycin	2.0	2.0	2.0	2.0	2.0

DF, diclofenac sodium; D, domperidone; GTN, glyceryl trinitrate; VP, verapamil; MF, metformin.

#### 5.5. The effect of tested compounds on the expression levels of efflux genes by qRT-PCR

**Fig 6:**
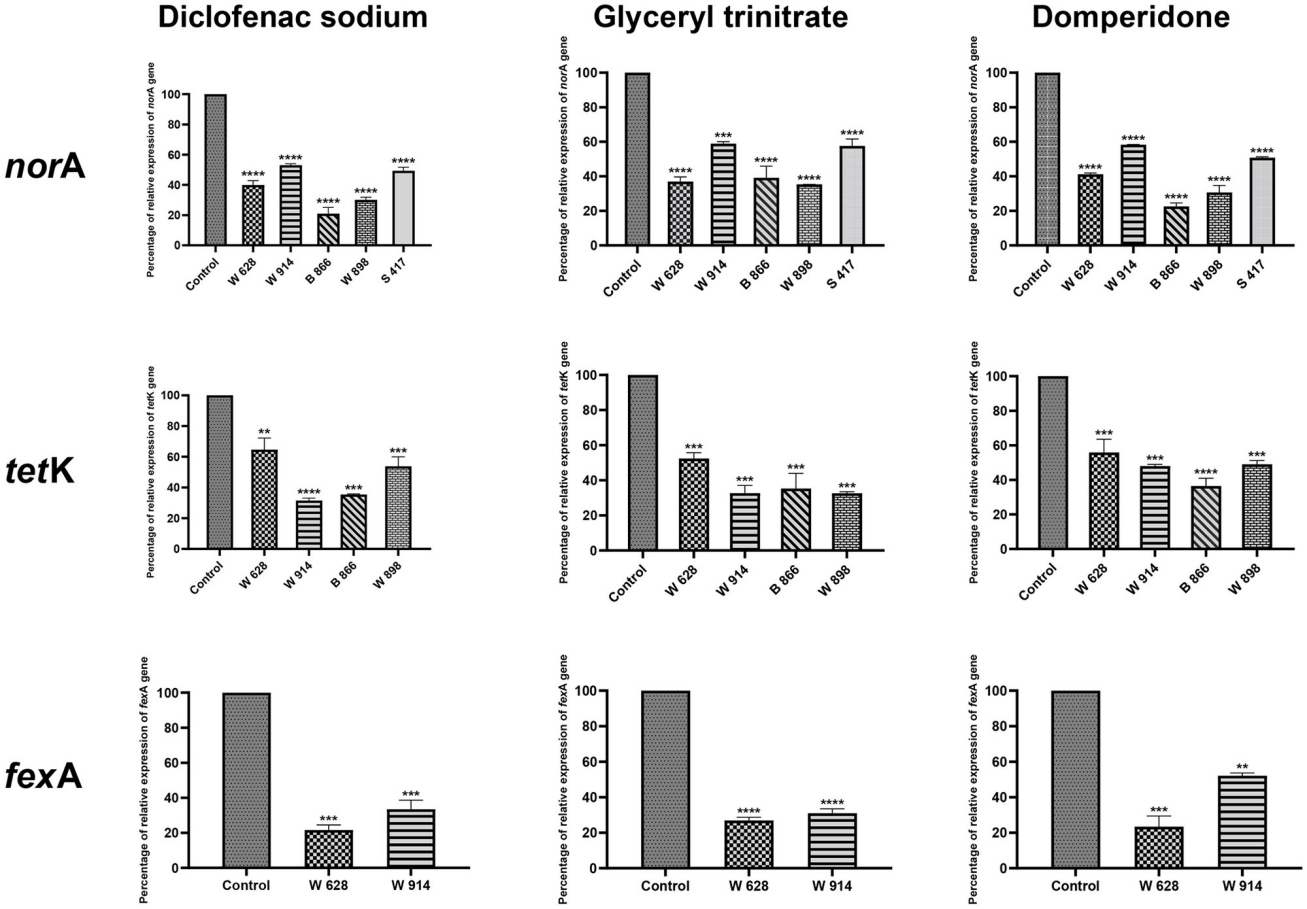
Down-regulation of *nor*A, *tet*K and *fex*A genes of *S*. *aureus* by diclofenac sodium, glyceryl trinitrate and domperidone which all produced significant reduction in the expression levels of these genes. The data shown represent the means ± standard errors. One Way ANOVA test followed by Dunnett’s Multiple Comparison test was used for the statistical analysis.

To ensure the EPI activity of the tested drugs, qRT-PCR was carried out. Five isolates that their efflux significantly reduced by the tested drugs were selected for qRT-PCR to investigate whether diclofenac sodium, domperidone and GTN affect the expression levels of the efflux genes (*nor*A, *fex*A and *tet*K) or not. Only diclofenac sodium, domperidone and GTN were tested because they showed significant EPI activities in EtBr-Cartwheel method, the efflux assay and decreased the MICs of tested antibiotics. The relative expression levels of *nor*A, *fex*A and *tet*K genes were significantly decreased by the tested drugs as shown in Fig 6.

Diclofenac sodium showed significant down-regulation of efflux genes that ranged from 46.97% to 79.01% for *nor*A gene, 30.01% to 53.03% for *tet*K gene and 46.97% to 50.68% for *fex*A gene. In addition, domperidone showed significant down-regulation that ranged from 41.71% to 77.41% for *nor*A gene, 30.74% to 58.29% for *tet*K gene and 41.71% to 49.2% for *fex*A gene. Moreover, GTN showed significant down-regulation that ranged from 41.09% to 64.68% for *nor*A gene, 39.13% to 58.91% for *tet*K gene and 41.09% to 42.42% for *fex*A gene. Diclofenac sodium is more potent inhibitor than domperidone and GTN against both *nor*A and *fex*A genes, while, GTN is more potent inhibitor than domperidone and diclofenac against *tet*K gene.

The significance of the efflux inhibitory activities of diclofenac sodium, domperidone and glyceryl trinitrate on the expression of efflux genes (*tet*K, *nor*A and *fex*A) were all found statistically significant (P values *<0*.*05* and *<0*.*001*).

## Discussion

*Staphylococcus aureus* is one of the most common causes of morbidity and mortality worldwide. It causes many diseases ranging from uncomplicated skin infections to more severe, invasive infections as fatal sepsis and pneumonia [54]. The emerging antimicrobial resistance of *S*. *aureus* make it one of the most common causes of the nosocomial infections [55]. Therefore, there is an urgent need for developing effective anti-staphylococcal therapy. The treatment of *S*. *aureus* infections remains challenging due to the emergence of MDR strains [56]. In addition, it can develop the resistance to different antibiotics by different mechanisms [2]. Therefore, rapid and accurate determination of antimicrobial resistance pattern and the underlying mechanisms have great importance [57].

In the current study, relatively high resistance rates were observed which may be due to the misuse of antibiotics in hospitals and community. In addition, the MDR was observed in 37.3% of the total isolates which is considered a relatively high percentage. Fortunately, our MDR rate is still lower than those reported by Tahaei *et al*. [58] and Abbas *et al*. [59]. Moreover, it is not strange that our isolates showed high resistance rates to β-lactams, which agree with Goudarzi *et al*. [60] study, as most of them are commonly prescribed and available for use in Egypt. Moreover, our Imipenem resistance rate is found higher than that of Gitau *et al*. [61] which may be because of the high prevalence of MRSA in current study. Moreover, our isolates showed low macrolides and lincosamides resistance rates, as these antibiotics aren’t prescribed widely in Egypt. Fortunately, our isolates didn’t show any resistance to vancomycin, linezolid or nitrofurantoin, which agree with Tahaei *et al*. [58] study. This may be due to their limited use.

The phenotypic detection of efflux activity was confirmed by the genotypic investigation of efflux genes using PCR. The current prevalence rates of *msr*A and *tet*K are found to be lower than that reported by Ceballos and his colleagues [62]. While, the current *fex*A prevalence rate was found to be higher than Ceballos and his colleagues study [62]. In addition, the present *nor*A rate is found to be higher than those of Hassanzadeh *et al*. [63] and Baiomy *et al*. [64]. Furthermore, the results of antibiotic susceptibility were compared with those of PCR using statistical analysis which revealed that the distribution of efflux genes regarding the phenotypic antibiotic resistance was statistically non-significant as that reported by Bissong and Ateba [65] study.

The efflux abilities of the isolates were detected qualitatively by EtBr-Cartwheel method. In our study, the active efflux activity was observed in 65.3% of *S*. *aureus* isolates which was less than that reported by Baiomy *et al*. [64] and Rana *et al*. [66], who reported the active efflux in all tested *S*. *aureus* isolates.

Efflux pump plays an important role in the MDR of *S*. *aureus*; therefore, it is vital to search for agents having an efflux inhibitory activity and the anti-efflux strategy is a promising option to treat the bacterial infections [67]. Thus, the repurposing of the drugs as the FDA-approved ones as it saves the costs and shortens the long time needed for the development of new antimicrobials [68]. This concept has been applied recently in many studies [59, 64, 69–74].

Therefore, in our study, the FDA-approved drugs as diclofenac sodium, domperidone, GTN and metformin were investigated for their EPI activities against *S*. *aureus* at their 1/8 MICs. Similarly, many previous studies have used the 1/4 or 1/8 of the MIC of the tested compounds [59, 64, 72–74]. Due to the use of 1/8 MIC of the tested compounds that had no effect on the bacterial growth, the possibility of the emergence of resistant mutants is much less than that in case of antibiotics that exert a stress on the bacterial growth. However, future studies are needed to confirm these results. In our study, the effective concentrations that produce an EPI action of verapamil were 8, 64, 150 and 187.5 μg/mL, of domperidone were 750, 875 and 1250 μg/mL, of GTN were 125 μg/mL, of diclofenac sodium was 48.8 μg/mL and of metformin was 1250 μg/mL. Therefore, diclofenac sodium followed by GTN (produced the highest EPI action with lower concentrations) were more potent than verapamil, domperidone and metformin (produced their EPI actions at higher concentrations).

The inhibitory effects of these drugs on the efflux were determined using qualitative and quantitative assays. Both methods showed that diclofenac sodium, domperidone and GTN were more potent than verapamil, while, metformin was the less potent. Also, the MICs of 6 antibiotics, that *S*. *aureus* resists them by efflux pumps (ciprofloxacin, doxycycline, chloramphenicol, azithromycin, erythromycin and clindamycin) were determined in the absence and the presence of tested drugs. It was found that the MICs of these antibiotics were decreased in the presence of diclofenac sodium, domperidone and GTN to values lower than those of verapamil and metformin which confirmed the results of efflux assay. Moreover, MICs of isolates that didn’t harbor the efflux genes didn’t decrease in the presence of tested drugs. Conversely, the study of Baiomy *et al*. [64] showed that metformin showed much higher activity in reducing the MICs than verapamil.

Domperidone is a safe and effective drug that can be used as EPI. Abdel-Halim *et al*. [27] suggested that domperidone-antibiotic combination can be used clinically for the treatment of MDR *E*. *coli* infections as it inhibited its efflux pump. However, Baiomy *et al*. [64] study found that domperidone had no effect on the efflux in *S*. *aureus*.

Metformin is one of the most commonly used hypoglycemic drugs that has been found to have EPI activity and was able to synergize the activity of some antibiotics. According to Baiomy *et al*. [64], metformin showed high EPI activity against *S*. *aureus*, while our study revealed that it had the low EPI activity. This difference may be due to using different clinical isolates from different sources in the two studies. In addition, only 2 isolates were screened in Baiomy *et al*. [64] study, while in our study, 72 isolates were tested. Furthermore, Baiomy *et al*. [64] study tested the EPI activities by two methods; EtBr-Cartwheel method and determining the decrease in the antibiotics MICs, while, our study used four methods in which our results were confirmed by RT-PCR unlike Baiomy *et al*. [64] study. In another study; Abbas *et al*. [73] study, metformin significantly reduced tetracycline and ciprofloxacin MICs in MDR of *Klebsiella pneumonia* isolates.

Several studies investigated the effect of FDA approved drugs on microbial virulence or Quorum sensing (QS). For example, Abbas and coworkers [59] showed that diclofenac sodium was able to inhibit the virulence of *S*. *aureus*. In addition, it was reported that GTN reduced several virulence factors in *S*. *aureus*, *Pseudomonas aeruginosa* and *Serratia marcescens* [32, 75, 76]. To our knowledge, our study is the first study that investigate the EPI activity of diclofenac, domperidone and GTN.

The efflux inhibitory activities of diclofenac, domperidone and GTN were further confirmed by investigating their effects on the expression levels of the efflux genes, *tet*K, *fex*A and *nor*A, by qRT-PCR. According to qRT-PCR results, diclofenac sodium is found to be more efficient in decreasing the expression levels of both *nor*A and *fex*A genes than domperidone and GTN, while, GTN is found to be more efficient in decreasing the expression levels of *tet*K gene than domperidone and diclofenac. Therefore, it is possible that the tested drugs inhibited the efflux pumps directly as well as repressed the transcription of the tested efflux genes. In a similar manner, Abbas *et al*. [59] study showed that diclofenac sodium significantly down-regulated the expression of *S*. *aureus* virulence genes, while, El-Ganiny *et al*. [74] study showed that domperidone significantly down-regulated *S*. *aureus* virulence genes.

Some studies showed that some compounds other than FDA-approved drugs can be used as EPIs against *S*. *aureus*. For example, the study of de Morais Oliveira-Tintino *et al*. [77] revealed that 1,8-naphthyridine sulfonamide derivatives possessed potential Tet(K) and MsrA efflux pump inhibitory activities. In addition, the natural product, sophoraflavanone G showed a potential *in vitro* and *in vivo* antibacterial activity and can be used as antibiotic-potentiating agent against *S*. *aureus* [78]. Moreover, the computational study of Ghosh *et al*. [79] suggested that cathinone had a good electrophilicity and ionization potential which enabled it to interact with most efflux pumps. Furthermore, Kim *et al*. [34] found that some piperic acid and 4-ethylpiperic acid synthetic amino acid amides could inhibit the efflux activity in *S*. *aureus* and enhance ciprofloxacin activity. In addition, Copper nanoparticles had been found to have a remarkable EPI and anti-biofilm activities in *S*. *aureus* [80]. Several studies suggested that some natural plant-derived compounds can be used as effective and less toxic EPIs in combination with antibiotics against *S*. *aureus* resistant strains [81–84].

*Staphylococcus aureus* is a major skin pathogen. As a result, the compounds tested in our study can be used topically in the treatment of localized skin infections even at high concentrations and thus their toxic effects will be minimum. For example, GTN is topically to treat the anal fissures at a concentration of 0.4% [29, 30]. In our study, GTN produced its effect at a much lower concentration (0.1%).

## Conclusion

Efflux plays a major role in the antibiotic resistance of *S*. *aureus*. Therefore, inhibiting efflux pumps could restore the activity of antibiotics. In addition, the use of FDA-approved drugs as EPIs is a promising strategy that can save time, money and efforts. In our study, diclofenac sodium, GTN and domperidone showed potent EPI activities and synergized the antibiotics against *S*. *aureus*. Thus, they can be used in combination with antibiotics for the treatment of the topical infections caused by *S*. *aureus*. In addition, these compounds may be structurally optimized in the future and be the leading ones for less toxic and more safe EPIs. However, further *in vivo* studies are required to confirm their activity and clinical efficacy.

## Supporting information

S1 Table Zone diameters (cm) of total isolates (n = 209) against tested antibiotics. No., the isolate number, W, isolate source is wound; B, isolate source is burn; U, isolate source is urine; BL, isolate source is blood; S, isolate source is sputum; E, isolate source is Endotracheal aspirate; P, penicillin G; OX, oxacillin; FOX, cefoxitin; AMC, amoxicillin / clavulanic acid; SAM, ampicillin / sulbactam; FEP, cefepime; CXM, ceforuxime; CFP, cefoperazone; IMP, imipenem; E, erythromycin; AZM, azithromycin; DA, clindamycin; C, chloramphenicol; AMK, amikacin; CN, gentamicin; RA, rifampin; SXT, sulphamethoxazole / trimethoprim; DO, doxycycline; NF, nitrofurantoin; LZ, linezolid; NOR, norfloxacin; CIP, ciprofloxacin.
(DOCX)

S2 Table Zone diameter breakpoints of tested antibiotics according to CLSI (2012). (DOCX)

S3 Table Resistance patterns in the total isolates (n = 209). R, resistant isolates; I, intermediate resistant isolate; S, sensitive isolates, No., number; %, percentage.
(DOCX)

S4 Table The resistance profile and the source of the selected *S*. *aureus* isolates (n = 72). W, wound is the isolate source; B, burn is the isolate source; U, urine is the isolate source; S, sputum is the isolate source; E, Endotracheal aspirate is the isolate source; P, penicillin G; OX, oxacillin; AMC, amoxicillin / clavulanic acid; SAM, ampicillin / sulbactam; FEP, cefepime; CXM, ceforuxime; CFP, cefoperazone; IMP, imipenem; E, erythromycin; AZM, azithromycin; DA, clindamycin; C, chloramphenicol; AMK, amikacin; CN, gentamicin; RA, rifampin; SXT, sulphamethoxazole / trimethoprim; DO, doxycycline; NOR, norfloxacin; CIP, ciprofloxacin. The antibiotics, mentioned in each row, are those to which each isolate was resistant.
(DOCX)

S5 Table Number of antibiotic resistant isolates that harbored the efflux genes from which the statistical analysis of the correlations was made. * The number shown in brackets beside each antibiotic represents the number of resistant isolates to that antibiotic.
(DOCX)

S6 Table Results of EtBr-Cartwheel method and the prevalence of efflux genes in the selected isolates (n = 72). No., the isolate number; P, positive efflux isolate; N, negative efflux isolate; I, intermediate efflux isolate; W, isolated from wound; U, isolated from urine; B, isolated from burn; E, isolated from endotracheal aspirate; S, isolated from sputum; +, gene present; -, gene absent.
(DOCX)

S7 Table The profile of the selected isolates (n = 26) for further analysis of efflux and testing the activity of tested drugs. No., isolate number; (+), positive for the gene; (-), negative for the gene; EtBrCW, EtBr Cart-Wheel test.
(DOCX)

S8 Table Qualitative detection of isolates efflux in the absence of tested compounds by Cart-Wheel method using AO. No., the isolate number; Conc., concentration; AO, acridine orange; +, fluorescence; ++, high fluorescence; +++, very high fluorescence.
(DOCX)

S9 Table The inhibitory effect of diclofenac sodium on the efflux activity by Cart-Wheel method using AO. Conc., concentration; AO, acridine orange; +, fluorescence; ++, high fluorescence; +++, very high fluorescence.
(DOCX)

S10 Table The inhibitory effect of verapamil on the efflux activity by Cart-Wheel method using AO. Conc., concentration; AO, acridine orange; +, fluorescence; ++, high fluorescence; +++, very high fluorescence.
(DOCX)

S11 Table The inhibitory effect of domperidone on the efflux activity by Cart-Wheel method using AO. Conc., concentration; AO, acridine orange; +, fluorescence; ++, high fluorescence; +++, very high fluorescence.
(DOCX)

S12 Table The inhibitory effect of GTN on the efflux activity by Cart-Wheel method using AO. Conc., concentration; AO, acridine orange; +, fluorescence; ++, high fluorescence; +++, very high fluorescence.
(DOCX)

S13 Table The inhibitory effect of metformin on the efflux activity by Cart-Wheel method using AO. Conc., concentration; AO, acridine orange; +, fluorescence; ++, high fluorescence; +++, very high fluorescence.
(DOCX)

S14 Table MICs (μg/ml) of ciprofloxacin (CIP) and chloramphenicol (C) alone and in the presence of the tested compounds. VP, verapamil; GTN, glyceryl trinitrate; D, domperidone; MF, metformin; DF, diclofenac sodium; ND, no decrease in MIC.
(DOCX)

S15 Table MICs (μg/ml) of doxycycline (DO) and azithromycin (AZM) alone and in the presence of the tested compounds. VP, verapamil; GTN, glyceryl trinitrate; D, domperidone; MF, metformin; DF, diclofenac sodium; ND, no decrease in MIC.
(DOCX)

S16 Table MICs (μg/ml) of erythromycin (EM) and clindamycin (DA) alone and in the presence of the tested compounds. VP, verapamil; GTN, glyceryl trinitrate; D, domperidone; MF, metformin; DF, diclofenac sodium; ND, no decrease in MIC.
(DOCX)

S1 Raw images **(A) Original uncropped and unadjusted image of *fex*A and *msr*A genes**. M; DNA ladder marker (3000 bp), Lanes 5–12; *fex*A gene (1272 bp), Lane 4; *msr*A gene (163 bp) and lanes 1–3; negative *msr*A isolates. **(B) Original uncropped and unadjusted image of *nor*A gene**. M; DNA ladder marker (3000 bp), Lanes 1–14, 16–23, 26; *nor*A gene (620 bp) and Lanes 15, 24, 25; negative *nor*A isolates. **(C) Original uncropped and unadjusted image of *tet*K gene**. M; DNA ladder marker (3000 bp), Lanes 2–4, 7–12, 14, 16–20; *tet*K gene (1159 bp), Lanes 1, 5, 6, 13, 15; contained negative *tet*K isolates and Lanes 21–26; negative results of another detected gene.
(PDF)
