# Supplementary material for: Promising FDA-approved drugs with efflux pump inhibitory activities against clinical isolates of Staphylococcus aureus
Source: PLoS One. 2022 Jul 29;17(7):e0272417. doi: 10.1371/journal.pone.0272417 (PMC9337675; doi:10.1371/journal.pone.0272417)
Supplement: S1 Table — No., the isolate number, W, isolate source is wound; B, isolate source is burn; U, isolate source is urine; BL, isolate source is blood; S, isolate source is sputum; E, isolate source is Endotracheal aspirate; P, penicillin G; OX, oxacillin; FOX, cefoxitin; AMC, amoxicillin / clavulanic acid; SAM, ampicillin / sulbactam; FEP, cefepime; CXM, ceforuxime; CFP, cefoperazone; IMP, imipenem; E, erythromycin; AZM, azithromycin; DA, clindamycin; C, chloramphenicol; AMK, amikacin; CN, gentamicin; RA, rifampin; SXT, sulphamethoxazole / trimethoprim; DO, doxycycline; NF, nitrofurantoin; LZ, linezolid; NOR, norfloxacin; CIP, ciprofloxacin. (DOCX) [file pone.0272417.s001.docx]

**Supplementary Table 1.** **Zone diameters (cm) of total isolates (n=209) against tested antibiotics**

| **No.** | **Isolate code** | **P** | **OX** | **FOX** | **AMC** | **SAM** | **FEP** | **CXM** | **CEP** | **IPM** | **E** | **AZM** | **DA** | **C** | **AMK** | **CN** | **RA** | **SXT** | **DO** | **NF** | **LZ** | **NOR** | **CIP** |
| --- | --- | --- | --- | --- | --- | --- | --- | --- | --- | --- | --- | --- | --- | --- | --- | --- | --- | --- | --- | --- | --- | --- | --- |
| **1** | **W 822** | 0 | 0 | 1 | 1.2 | 0 | 0 | 1 | 0 | 0 | 0 | 0 | 0 | 0.9 | 1 | 0.9 | 2.1 | 3 | 1 | 2.6 | 3.8 | 0.6 | 0.9 |
| **2** | **W 823** | 0 | 0 | 0 | 1 | 0 | 0 | 0 | 0 | 0 | 0 | 0 | 0 | 1 | 0.9 | 0 | 2 | 2.9 | 0.8 | 2 | 3.6 | 0 | 0 |
| **3** | **W 871** | 0 | 0 | 0 | 0.9 | 0 | 0 | 1.4 | 0 | 0 | 0 | 0 | 0 | 0.8 | 1.4 | 0 | 3 | 4.8 | 0 | 3.5 | 5.4 | 0 | 0 |
| **4** | **W 898** | 0 | 0 | 0 | 1.2 | 0 | 0 | 1.6 | 0 | 0 | 0 | 0 | 0 | 0.8 | 1.1 | 0 | 3 | 4.3 | 0.9 | 3.4 | 5.3 | 0 | 0 |
| **5** | **W 904** | 0 | 0 | 0 | 1.5 | 0 | 0 | 0 | 0 | 0.8 | 0 | 0 | 0 | 4.6 | 1 | 0 | 2.3 | 4.8 | 1 | 3.8 | 5.2 | 0 | 0 |
| **6** | **W 881** | 0 | 0 | 0 | 1.3 | 0 | 0 | 1.4 | 0 | 0 | 0 | 0 | 0 | 3.5 | 1.4 | 0 | 2.3 | 3.8 | 1 | 3 | 5.2 | 0 | 0 |
| **7** | **W 428** | 0 | 0 | 0 | 1.5 | 0 | 0 | 0 | 0 | 0 | 0 | 0 | 0 | 3 | 0.8 | 0 | 2.2 | 3.5 | 0 | 2.6 | 4.7 | 0 | 0 |
| **8** | **W 429** | 0 | 0 | 0 | 1.4 | 1.9 | 0 | 0 | 0 | 1 | 0 | 0 | 0 | 0.9 | 0 | 0 | 1.8 | 1.5 | 0 | 2.6 | 3.8 | 0 | 0 |
| **9** | **W 869** | 0 | 0 | 0 | 0.8 | 0 | 0 | 0 | 0 | 0 | 0 | 0 | 0 | 2.8 | 1.5 | 0.9 | 2 | 3.5 | 0.8 | 2.5 | 4 | 0 | 0 |
| **10** | **W 877** | 0 | 0 | 0 | 0.9 | 0 | 0 | 0 | 0 | 0 | 3.1 | 2.7 | 3.1 | 0.8 | 0 | 0 | 1.5 | 3.1 | 0.9 | 2.3 | 3.3 | 0 | 0 |
| **11** | **W 820** | 0 | 0 | 1.2 | 1.4 | 1.4 | 0 | 1.8 | 0 | 0 | 0 | 0 | 0 | 4.2 | 0.9 | 0 | 2.7 | 4.5 | 1 | 3.2 | 5.4 | 0 | 0 |
| **12** | **W 873** | 0 | 0 | 0 | 0.9 | 0 | 0 | 0 | 0 | 0 | 3.7 | 3.6 | 3.2 | 0.9 | 0 | 0 | 1.9 | 3.4 | 0 | 2.5 | 4.5 | 0 | 0 |
| **13** | **W 887** | 0 | 0 | 0 | 1 | 0 | 0 | 0 | 0 | 0.8 | 3 | 2.8 | 3.3 | 3 | 1 | 0 | 1.5 | 2.7 | 0 | 2.3 | 3.7 | 0 | 0 |
| **14** | **W 446** | 0 | 0 | 1.1 | 1.1 | 2 | 0 | 0 | 0 | 0 | 3.4 | 3 | 3.2 | 2.5 | 1.1 | 0 | 1.8 | 0 | 1.1 | 2.2 | 4 | 1 | 0 |
| **15** | **W 916** | 0 | 0 | 0 | 1 | 0 | 0 | 0 | 0 | 0 | 3.8 | 3.8 | 3.9 | 3.4 | 1.1 | 0 | 2 | 3.8 | 1 | 2.6 | 4.5 | 0 | 0 |
| **16** | **W 915** | 0 | 0 | 0 | 0.9 | 1.4 | 0 | 0 | 0 | 0 | 4.2 | 4.2 | 4.4 | 4 | 1.6 | 0 | 2.4 | 4.3 | 0.8 | 3.4 | 5.4 | 0 | 0 |
| **17** | **W 752** | 0 | 0 | 0 | 0 | 0 | 0 | 0 | 0 | 4.5 | 1.2 | 1.3 | 3.3 | 3.2 | 2.9 | 1.3 | 4.1 | 1.5 | 3 | 2.5 | 4 | 0 | 0 |
| **18** | **W 436** | 2.5 | 2.4 | 3.6 | 3.4 | 2.8 | 2.6 | 1 | 2.7 | 5.6 | 0 | 0 | 0 | 1 | 0.8 | 0 | 2.5 | 3.8 | 1.1 | 2.5 | 4.8 | 0 | 0 |
| **19** | **W 422** | 1.8 | 0 | 2.2 | 2.4 | 1.5 | 1.8 | 0 | 3 | 5 | 3.8 | 3.3 | 3.2 | 2.8 | 1.4 | 1 | 2.1 | 3.7 | 1 | 2.6 | 3.8 | 0 | 1.6 |
| **20** | **W 2** | 1.6 | 0 | 1.8 | 2.6 | 1.5 | 1.7 | 0 | 2 | 4 | 0 | 0 | 3.4 | 3.2 | 1.4 | 1.2 | 3.6 | 3.5 | 1.3 | 2.4 | 4 | 2.8 | 2.7 |
| **21** | **W 914** | 2.6 | 1.6 | 2.8 | 3.1 | 2.7 | 2.4 | 0 | 2.7 | 4.4 | 0 | 0 | 0 | 1 | 3.8 | 3 | 4.1 | 4 | 0 | 2.4 | 4.8 | 0 | 0 |
| **22** | **W 439** | 1.5 | 0 | 1.1 | 3.2 | 3.2 | 2.4 | 0 | 2.6 | 3.3 | 1.1 | 1.2 | 3.8 | 4 | 1.2 | 0 | 4.4 | 1.8 | 3.2 | 3.2 | 5 | 3.6 | 3.8 |
| **23** | **W 419** | 1.2 | 0 | 1.5 | 1.6 | 0.9 | 1.4 | 1 | 1.6 | 3.2 | 3.3 | 3.3 | 3.1 | 3 | 2.3 | 2.2 | 3.8 | 3.5 | 2.8 | 2.4 | 3.9 | 2.5 | 2.8 |
| **24** | **W 894** | 1.1 | 0 | 1.6 | 1.8 | 1.7 | 1.5 | 0 | 1.9 | 4.4 | 3.3 | 3 | 3 | 1.2 | 2.5 | 2.6 | 3.5 | 2.8 | 1.1 | 2.4 | 4 | 2.8 | 2.9 |
| **25** | **W 504** | 2.2 | 0 | 2.1 | 2.6 | 2 | 2.3 | 0 | 2.5 | 4 | 3.5 | 3.4 | 3.5 | 3 | 1.2 | 1.1 | 4 | 3.5 | 1 | 2.6 | 3.8 | 2.3 | 2.7 |
| **26** | **W 879** | 2.6 | 0 | 2 | 2.6 | 2.5 | 1.8 | 0 | 2.5 | 3.5 | 3.2 | 3.2 | 3.2 | 2.8 | 1.1 | 1.1 | 3.5 | 3.5 | 2.6 | 2.3 | 3.8 | 0.9 | 2 |
| **27** | **W 518** | 1 | 0 | 1.9 | 2.3 | 2 | 1.7 | 0 | 2 | 3.8 | 0.8 | 0.7 | 3.4 | 2.5 | 2.5 | 2.4 | 3.7 | 2.5 | 1 | 2.3 | 3.8 | 2.7 | 2.7 |
| **28** | **W 540** | 2 | 0 | 2 | 2.5 | 2 | 2 | 0 | 2.2 | 3.9 | 3.5 | 3.5 | 2.9 | 3 | 1.2 | 1 | 4 | 3.6 | 1.2 | 2.6 | 4 | 3.2 | 2.7 |
| **29** | **W 3** | 1.5 | 1.1 | 1.9 | 2.2 | 2 | 1.9 | 0.9 | 2.6 | 3.8 | 0.9 | 1 | 2.9 | 2.6 | 1.5 | 1.3 | 3.5 | 2.8 | 2.4 | 2.3 | 3.6 | 0 | 0 |
| **30** | **W 505** | 2 | 1.2 | 2.1 | 2.6 | 2.4 | 2.1 | 0 | 2.1 | 3.7 | 3.3 | 3.3 | 1.9 | 1.1 | 2.1 | 2.2 | 3 | 3.8 | 0 | 2.3 | 3.6 | 0.8 | 2.1 |
| **31** | **W 413** | 1 | 0 | 0 | 1.8 | 1 | 1.6 | 0 | 1.8 | 3.5 | 3.6 | 3.4 | 3.4 | 3.3 | 2.4 | 2.8 | 3.6 | 2 | 2.5 | 2.6 | 3.7 | 2.2 | 2.2 |
| **32** | **W 628** | 1.5 | 1 | 1.9 | 2.3 | 1.6 | 1.9 | 0.9 | 1.9 | 3.8 | 3.1 | 2.9 | 3.1 | 1 | 2.2 | 2.3 | 3.3 | 3 | 0.7 | 2.3 | 3.6 | 0 | 0 |
| **33** | **W 403** | 1.5 | 0 | 2.1 | 2.1 | 1.7 | 1.9 | 0.7 | 1.8 | 3.3 | 3.2 | 3 | 3 | 2.6 | 1.2 | 1 | 3.3 | 3.8 | 1.3 | 2.2 | 3.8 | 2.7 | 3 |
| **34** | **W 359** | 1 | 0 | 1.8 | 1.4 | 1.6 | 1.5 | 0 | 2 | 3.7 | 3.1 | 3.2 | 3.4 | 3.4 | 2.8 | 3 | 3.7 | 2.6 | 1.2 | 2.5 | 3.8 | 3.1 | 3.2 |
| **35** | **W 536** | 2.2 | 1 | 2.1 | 2.5 | 2 | 1.9 | 0 | 3.2 | 3.6 | 2.9 | 3 | 3 | 2.7 | 2.2 | 2.7 | 3.5 | 3.3 | 1.1 | 2.3 | 3.6 | 2.1 | 2.4 |
| **36** | **W 365** | 1.3 | 1.6 | 1.9 | 2.3 | 1.5 | 2 | 0.7 | 1.8 | 4.2 | 0 | 0 | 3 | 2.5 | 2.5 | 2.5 | 3.1 | 0 | 1.8 | 2 | 3.5 | 2.7 | 2.8 |
| **37** | **W 438** | 2 | 0 | 2.1 | 2.6 | 2.3 | 1.9 | 0 | 2.1 | 3.6 | 3.6 | 3.1 | 3.2 | 3.1 | 1.6 | 0 | 3.8 | 3.5 | 1.4 | 2.6 | 3.9 | 2.7 | 3 |
| **38** | **W 450** | 1.5 | 0 | 2.1 | 2.4 | 1.9 | 1.7 | 0 | 1.9 | 3.7 | 3.1 | 2.8 | 3.4 | 1 | 2.3 | 2.3 | 3.2 | 2.9 | 1.3 | 2.4 | 3.5 | 2.7 | 2.6 |
| **39** | **W 885** | 2.2 | 0 | 2.2 | 2.8 | 2.4 | 2.2 | 2 | 2.6 | 4.4 | 3.8 | 3.8 | 3.6 | 3.4 | 3 | 3 | 4.4 | 4 | 1.4 | 2.6 | 4.4 | 0.6 | 0.6 |
| **40** | **W 932** | 1.2 | 0 | 1.7 | 1.8 | 1.3 | 2.2 | 0 | 1.9 | 5 | 3 | 3 | 3 | 3 | 2.7 | 2.7 | 3.3 | 1.9 | 2.9 | 2.6 | 3.7 | 3 | 3 |
| **41** | **W 876** | 2.8 | 0 | 2.1 | 2.6 | 2.6 | 2.2 | 0 | 2.3 | 3.7 | 3.2 | 3.6 | 3.6 | 3 | 3 | 2.8 | 4.2 | 3.3 | 2.6 | 2.4 | 4.4 | 0.8 | 2.4 |

**Supplementary Table 1. Continued**

| **No.** | **Isolate code** | **P** | **OX** | **FOX** | **AMC** | **SAM** | **FEP** | **CXM** | **CEP** | **IPM** | **E** | **AZM** | **DA** | **C** | **AMK** | **CN** | **RA** | **SXT** | **DO** | **NF** | **LZ** | **NOR** | **CIP** |
| --- | --- | --- | --- | --- | --- | --- | --- | --- | --- | --- | --- | --- | --- | --- | --- | --- | --- | --- | --- | --- | --- | --- | --- |
| **42** | **W 629** | 2.6 | 0.9 | 2.6 | 3.4 | 3.2 | 2.4 | 1.8 | 2.5 | 4.5 | 4.4 | 4.4 | 4 | 3.8 | 3.6 | 1.8 | 4.6 | 4.4 | 2 | 3.6 | 4.3 | 0.8 | 0.8 |
| **43** | **W 907** | 0 | 0 | 1.9 | 1.9 | 1.6 | 1.6 | 0 | 1.6 | 3.2 | 3.9 | 3.6 | 3.5 | 1.4 | 2.8 | 3 | 4 | 3.4 | 1.5 | 2.7 | 4.2 | 3.1 | 3 |
| **44** | **W 817** | 1.2 | 0 | 2.1 | 2.1 | 1.5 | 1.4 | 0.9 | 1.9 | 3.5 | 3.1 | 3 | 3 | 2.5 | 2.7 | 2.7 | 3.3 | 2 | 2.2 | 2.4 | 3.3 | 3 | 3 |
| **45** | **W 379** | 1.5 | 1.9 | 1.8 | 2.3 | 1.9 | 2 | 0.9 | 1.9 | 5 | 0 | 0 | 2.8 | 2.8 | 1 | 0.7 | 3.3 | 3 | 1.8 | 2.1 | 4.2 | 1.9 | 2.1 |
| **46** | **W 870** | 2.6 | 0 | 2.1 | 2.5 | 2.2 | 1.8 | 0 | 2.1 | 3 | 3.2 | 3 | 3.2 | 3 | 1.8 | 2.8 | 4 | 3.4 | 2.6 | 2.3 | 3.8 | 1.3 | 1.8 |
| **47** | **W 636** | 1.6 | 0 | 2 | 2.8 | 2 | 2 | 0 | 2.2 | 4 | 3.6 | 3.1 | 1.7 | 3.1 | 2.8 | 2.6 | 2.6 | 3.6 | 1.5 | 2.5 | 4 | 2.6 | 2.7 |
| **48** | **W 822** | 2 | 0 | 1 | 2.9 | 3.6 | 2.3 | 0.7 | 2.5 | 4.4 | 3.5 | 3.5 | 3.8 | 3.5 | 2.5 | 1.4 | 3.8 | 3.7 | 1 | 2.7 | 4.4 | 2.8 | 3.1 |
| **49** | **W 824** | 2.2 | 0 | 2 | 2.6 | 1.7 | 2 | 1 | 2.4 | 5 | 3.2 | 2.9 | 2.8 | 2.8 | 2.5 | 2.3 | 3.1 | 1.9 | 2.5 | 3.5 | 3.5 | 2.5 | 2.6 |
| **50** | **W 519** | 2 | 0 | 2.1 | 2.6 | 2.4 | 1.9 | 0 | 2.6 | 4.2 | 3.9 | 3.6 | 3.7 | 3.2 | 2.6 | 2.6 | 3.8 | 3.9 | 2.6 | 2.4 | 4.3 | 2.8 | 2.8 |
| **51** | **W 523** | 1.6 | 0 | 2 | 2.3 | 2 | 1.7 | 0 | 2 | 3.4 | 3.1 | 3.2 | 3.2 | 2.8 | 2.1 | 2.8 | 3.6 | 3.5 | 2.5 | 2.6 | 3.9 | 3 | 2.6 |
| **52** | **W 529** | 2 | 0 | 1.5 | 2.5 | 1.8 | 1.8 | 0 | 2 | 3.5 | 3.5 | 3.2 | 3.2 | 3 | 2.3 | 2.4 | 3.5 | 3.4 | 1.5 | 2 | 3.9 | 2.5 | 2.7 |
| **53** | **W 527** | 1.2 | 0 | 3 | 2.4 | 2.1 | 2.6 | 2.8 | 2.1 | 4.6 | 3.1 | 3 | 3.3 | 2.8 | 2.4 | 2.6 | 3.6 | 3.5 | 3 | 2.5 | 3.8 | 3 | 3 |
| **54** | **W 373** | 1.5 | 0 | 1.1 | 2.1 | 2.2 | 1.8 | 0.9 | 1.9 | 3.7 | 3 | 3.1 | 3.4 | 3.2 | 2.5 | 1.4 | 3.4 | 3.5 | 2.4 | 2.5 | 3.3 | 3 | 3 |
| **55** | **W 362** | 1.8 | 0 | 1.4 | 2.5 | 2.1 | 2.5 | 0 | 2.2 | 5 | 2.3 | 2.7 | 3 | 2.5 | 2.2 | 2.2 | 2.5 | 2.9 | 2 | 2.4 | 3.3 | 2.6 | 2.8 |
| **56** | **W 408** | 1.3 | 0 | 1.8 | 2.2 | 2.6 | 1.8 | 1.5 | 2 | 3.5 | 3.5 | 3 | 3.3 | 3.4 | 2.5 | 2.1 | 3.8 | 3.8 | 2 | 2.1 | 4.5 | 2.5 | 3.4 |
| **57** | **W 821** | 2 | 1 | 3.2 | 2.7 | 2.1 | 2.3 | 1.3 | 2.3 | 4.7 | 2.8 | 2.7 | 2.9 | 2.8 | 1.7 | 1.8 | 3.2 | 3.3 | 2.6 | 2 | 3.6 | 2.7 | 2.6 |
| **58** | **W 816** | 2.6 | 1 | 2.2 | 3 | 2.6 | 2.3 | 0 | 2.6 | 4.4 | 3.7 | 3.7 | 3.4 | 3.5 | 2.4 | 1.3 | 4.1 | 2.6 | 1.3 | 3 | 4.6 | 2.9 | 3 |
| **59** | **W 246** | 2.6 | 1.5 | 2.4 | 2.8 | 2.2 | 2.1 | 0 | 2.1 | 4.4 | 3.8 | 3.6 | 3.1 | 3.4 | 1.1 | 0 | 4 | 3.3 | 1.5 | 2.6 | 4 | 2.8 | 2.5 |
| **60** | **W 625** | 1.8 | 0 | 2.2 | 2.8 | 2 | 2.1 | 0 | 2.3 | 4 | 3.3 | 3.3 | 3.3 | 3.3 | 2.4 | 2.6 | 3.8 | 3.8 | 1.8 | 2.8 | 4.1 | 2.3 | 3.8 |
| **61** | **W 882** | 2.3 | 0 | 2.2 | 3 | 2.4 | 2.2 | 0 | 2.2 | 4.1 | 3.8 | 3.7 | 3.7 | 1.4 | 2.4 | 2.6 | 4.1 | 3 | 1.4 | 2.8 | 4.6 | 2.8 | 3.2 |
| **62** | **W 889** | 1.8 | 1.4 | 2.1 | 2.4 | 3.4 | 1.7 | 0 | 2.2 | 4 | 3.6 | 3.8 | 3.4 | 3.4 | 2.6 | 2.8 | 4 | 1.8 | 2.3 | 2.6 | 3.8 | 2.8 | 2.3 |
| **63** | **W 416** | 1.3 | 0.8 | 2 | 2.3 | 1.9 | 2 | 1.5 | 2 | 3.7 | 3.1 | 3.1 | 3.3 | 2.8 | 2 | 2.2 | 3.5 | 3.6 | 2.6 | 2 | 3.7 | 2.5 | 2.7 |
| **64** | **W 890** | 2 | 1 | 2.4 | 2.9 | 2.8 | 2 | 2 | 2.4 | 4.2 | 4 | 4.2 | 4 | 0 | 3.3 | 3.2 | 4 | 3.2 | 1.4 | 3 | 4 | 3.6 | 2.8 |
| **65** | **W 507** | 2.4 | 1.8 | 2.8 | 3 | 2.6 | 2.2 | 0.7 | 2.6 | 4.2 | 1 | 0 | 3.4 | 3.2 | 3 | 2.8 | 4 | 3.7 | 2.8 | 2.6 | 4.2 | 3.2 | 3 |
| **66** | **W 512** | 2 | 1.4 | 2.6 | 2.6 | 2.2 | 2.3 | 0 | 2.6 | 4 | 1.1 | 1.2 | 3.2 | 2.7 | 2.2 | 2.2 | 3.5 | 2 | 2.4 | 2.5 | 3.7 | 2.7 | 3 |
| **67** | **W 369** | 1.6 | 0 | 2.2 | 2.3 | 1.8 | 2.1 | 0.9 | 1.9 | 3.9 | 3.3 | 3.3 | 3.2 | 3.4 | 2.6 | 1.3 | 3.6 | 3.6 | 3 | 2.6 | 4 | 3.2 | 3.2 |
| **68** | **W 380** | 1.1 | 0.9 | 3 | 2.3 | 1.9 | 2 | 1.5 | 1.9 | 4 | 2.9 | 2.6 | 2.7 | 2.6 | 2.7 | 2.7 | 3.1 | 3.6 | 2.4 | 2.4 | 3.1 | 3 | 2.9 |
| **69** | **W 364** | 1.5 | 1.1 | 1.6 | 2.5 | 1.5 | 2 | 0 | 2.6 | 4.5 | 3.8 | 3.4 | 3.5 | 3 | 2.7 | 2.8 | 3.7 | 1.2 | 3.6 | 2.4 | 4.2 | 3 | 3.4 |
| **70** | **W 368** | 2.8 | 1.6 | 1.7 | 2.8 | 2.3 | 1.7 | 1 | 2.2 | 3.8 | 3 | 3 | 2.7 | 2.8 | 1.6 | 0 | 3.3 | 2.7 | 2.8 | 2.3 | 4.2 | 2.4 | 2.3 |
| **71** | **W 241** | 3 | 1.8 | 2.6 | 3.2 | 2.8 | 2.2 | 2 | 2.6 | 4.4 | 3.6 | 3.5 | 3.4 | 3.2 | 1.4 | 1 | 3.7 | 3.8 | 1.2 | 2.3 | 4.2 | 2.8 | 2.8 |
| **72** | **W 908** | 2.6 | 2 | 2.8 | 3 | 2.4 | 2.6 | 2.2 | 2.6 | 4.6 | 0 | 0 | 3.2 | 2.4 | 2.8 | 2.8 | 3.9 | 2.2 | 2.6 | 2.4 | 4.2 | 2.4 | 2.6 |
| **73** | **W 878** | 2.2 | 1.4 | 2.4 | 3.1 | 2.4 | 2.4 | 0 | 2.3 | 4.2 | 3.4 | 3.4 | 3.4 | 0.9 | 2.5 | 2.6 | 4 | 2.6 | 1.4 | 2.6 | 4.4 | 2.4 | 3.1 |
| **74** | **W 884** | 3.6 | 2.4 | 2.4 | 3.2 | 3 | 2 | 1.5 | 3 | 4.2 | 4 | 4 | 3.8 | 3 | 1.4 | 0.8 | 4.2 | 3.8 | 3.3 | 2.6 | 4.2 | 1.2 | 1.8 |
| **75** | **W 366** | 1.5 | 1.5 | 2.3 | 2.3 | 2 | 7 | 0 | 2 | 4.7 | 0 | 0 | 3.3 | 2.8 | 2.5 | 1.4 | 3.4 | 2.4 | 2.6 | 2.5 | 4.2 | 2.8 | 5.2 |
| **76** | **W 508** | 2.1 | 1.3 | 2.2 | 2.8 | 2.2 | 2.4 | 0.8 | 2.3 | 3.9 | 2.1 | 1 | 3.1 | 2.6 | 2.2 | 2.6 | 3.5 | 1.6 | 2.7 | 1.8 | 4 | 2.2 | 2.7 |
| **77** | **W 509** | 2.6 | 1.6 | 3 | 3.2 | 2.6 | 3 | 0.8 | 2.8 | 5.4 | 3.2 | 1 | 3.4 | 3.2 | 2.4 | 3 | 3.4 | 3.6 | 2.8 | 2.5 | 3.8 | 3.3 | 2.6 |
| **78** | **W 517** | 2.6 | 1.2 | 2.8 | 2.6 | 3 | 3 | 1.8 | 3 | 4.4 | 1.2 | 1.6 | 4.2 | 3.8 | 3.4 | 3.4 | 4 | 2.2 | 2 | 2.8 | 4.8 | 3.8 | 4 |
| **79** | **W 506** | 2 | 1.6 | 3.2 | 2.9 | 2.3 | 2.6 | 1 | 2.4 | 4 | 3.4 | 3.4 | 3.2 | 3 | 2.2 | 2.4 | 3.4 | 3.1 | 2.6 | 2.3 | 4 | 2.4 | 2.7 |
| **80** | **W 631** | 2.3 | 2.1 | 3.6 | 3 | 2.3 | 2.8 | 1.4 | 2.5 | 4.2 | 3.8 | 3.4 | 3.8 | 3.8 | 2.5 | 2.6 | 4 | 4.4 | 3.1 | 2.6 | 4.6 | 2.7 | 3 |
| **81** | **W 635** | 2.2 | 1.4 | 2.4 | 2.8 | 2.4 | 2.1 | 2.2 | 2.4 | 4.6 | 4.2 | 3.7 | 3.6 | 3.4 | 2.4 | 1.3 | 4.2 | 4.2 | 1 | 2.6 | 4.6 | 3.4 | 3.6 |

**Supplementary Table 1. Continued**

| **No.** | **Isolate code** | **P** | **OX** | **FOX** | **AMC** | **SAM** | **FEP** | **CXM** | **CEP** | **IPM** | **E** | **AZM** | **DA** | **C** | **AMK** | **CN** | **RA** | **SXT** | **DO** | **NF** | **LZ** | **NOR** | **CIP** |
| --- | --- | --- | --- | --- | --- | --- | --- | --- | --- | --- | --- | --- | --- | --- | --- | --- | --- | --- | --- | --- | --- | --- | --- |
| **82** | **W 245** | 2.6 | 1.8 | 3.2 | 3 | 2.2 | 2.6 | 0.8 | 2.5 | 5.6 | 3.6 | 3.8 | 3.6 | 3.4 | 2.6 | 2.8 | 4 | 4 | 3 | 2.8 | 4.3 | 2.5 | 3 |
| **83** | **W 242** | 1.9 | 1.3 | 2.4 | 2.6 | 2.4 | 2 | 0.9 | 2.2 | 4.5 | 3.3 | 3.2 | 3.4 | 3.4 | 2.5 | 2.6 | 3.7 | 3.8 | 2.6 | 2.6 | 4 | 2.8 | 3 |
| **84** | **W 821** | 2.6 | 2.1 | 3.6 | 3.2 | 2 | 2.8 | 3 | 2.4 | 4 | 4 | 3.2 | 3.2 | 3 | 2.6 | 2.8 | 4.2 | 4.2 | 1.4 | 2.6 | 4 | 2.7 | 2.8 |
| **85** | **W 352** | 1.5 | 1.9 | 2.2 | 2.5 | 2.5 | 2 | 1.4 | 2.1 | 4.5 | 0 | 0 | 1.8 | 2.5 | 2 | 1.8 | 3 | 1.6 | 2 | 2.5 | 4.2 | 2.5 | 2.8 |
| **86** | **W 448** | 2.4 | 2 | 3.2 | 3.2 | 2.5 | 2.6 | 0.9 | 2.6 | 5 | 3.5 | 3.4 | 3.1 | 3 | 2.4 | 2.4 | 3.8 | 3.6 | 2.6 | 2.5 | 3.8 | 2.8 | 3 |
| **87** | **W 433** | 1.7 | 1.3 | 3 | 2.7 | 2.2 | 2.5 | 1.1 | 2.3 | 4.5 | 3.1 | 3 | 3 | 3 | 2.2 | 2.4 | 3.5 | 3.6 | 2.8 | 2.3 | 3.8 | 2.4 | 2.7 |
| **88** | **W 360** | 1.2 | 1.8 | 1.7 | 2.3 | 1.7 | 1.7 | 0 | 1.9 | 4.7 | 3.3 | 2.9 | 2.8 | 3 | 1.7 | 2.5 | 3.5 | 3.2 | 3 | 2.3 | 4.2 | 2.3 | 2.4 |
| **89** | **W 936** | 1.2 | 1.9 | 2.4 | 2.9 | 1.8 | 2 | 0 | 2.4 | 5.2 | 3.8 | 3.6 | 3.8 | 3.2 | 2.8 | 2.7 | 4 | 1.3 | 3.6 | 2.4 | 4.4 | 3.2 | 3.6 |
| **90** | **W 867** | 2 | 1.4 | 2 | 2 | 1.7 | 2 | 1.7 | 2.2 | 4 | 3.4 | 3.3 | 3.6 | 3.4 | 2.6 | 2.6 | 3.7 | 3.8 | 2.1 | 2.4 | 4.2 | 2.4 | 2.6 |
| **91** | **W 538** | 2.4 | 1.6 | 3.4 | 2.9 | 3.6 | 2.9 | 0.9 | 2.4 | 4.2 | 3.4 | 3.4 | 3.3 | 3 | 2.4 | 2.6 | 3.8 | 3.8 | 1.5 | 2.2 | 4.2 | 2.8 | 2.8 |
| **92** | **W 369** | 1.5 | 1.6 | 1.8 | 2.3 | 1.9 | 1.9 | 0.7 | 2 | 4.7 | 3.1 | 3 | 2.7 | 2.8 | 2.5 | 1.4 | 3.4 | 3 | 2.8 | 2.6 | 4 | 2.6 | 2.7 |
| **93** | **W 719** | 2.2 | 2 | 3 | 3 | 2.6 | 2.6 | 2.6 | 2.6 | 5.2 | 3.4 | 3.4 | 3.4 | 3.2 | 2.6 | 2.6 | 3.9 | 4 | 3 | 2.4 | 4.3 | 3.2 | 3.2 |
| **94** | **W 632** | 2.2 | 2 | 3 | 2.8 | 2.2 | 2.6 | 2.4 | 2.6 | 5 | 3.6 | 3.4 | 3.2 | 2.8 | 2.8 | 2.6 | 4 | 3.6 | 2.6 | 2.4 | 4 | 3.1 | 3 |
| **95** | **W 872** | 2.9 | 2 | 2.7 | 3.2 | 2.6 | 2.6 | 1.1 | 2.6 | 4.5 | 2 | 2.1 | 3 | 3.2 | 2.6 | 2.8 | 4 | 2.4 | 2.7 | 2.6 | 4.1 | 2.5 | 2.6 |
| **96** | **W 880** | 3 | 2.3 | 3 | 3.6 | 2.8 | 2.8 | 1.9 | 2.9 | 6 | 4.2 | 4.2 | 4 | 4 | 3.2 | 3.4 | 2.9 | 5.4 | 1.2 | 3 | 5 | 3.4 | 3.4 |
| **97** | **W 895** | 3 | 1.7 | 2.4 | 3.1 | 2.8 | 2.2 | 0 | 2.5 | 4.2 | 3 | 3.1 | 3.1 | 3 | 2.4 | 2.6 | 3.7 | 2.1 | 2.6 | 2 | 3.7 | 2.6 | 3 |
| **98** | **W 440** | 1.8 | 1.9 | 2.8 | 2.7 | 2 | 2.7 | 2.6 | 2.3 | 4.9 | 3.1 | 3.3 | 3 | 3.1 | 2.7 | 2.5 | 3.5 | 3.7 | 2.7 | 2.3 | 4.2 | 2.5 | 2.7 |
| **99** | **W 243** | 2.6 | 2.2 | 3 | 3 | 3.6 | 2.8 | 2.6 | 1.8 | 5 | 3.6 | 3.2 | 3.4 | 3.2 | 2.6 | 2.7 | 3.8 | 3.8 | 2.9 | 2.6 | 4.3 | 2.8 | 3 |
| **100** | **W 874** | 3 | 1.3 | 3.4 | 3.2 | 3.6 | 3 | 1.8 | 2.5 | 4.4 | 2.9 | 3.4 | 3.2 | 3.4 | 3 | 3.2 | 4.5 | 3.4 | 2.6 | 2.6 | 4.2 | 2.6 | 3 |
| **101** | **W 892** | 3.3 | 1.5 | 3 | 3.4 | 2.6 | 2.8 | 1.8 | 2.8 | 4.8 | 3.3 | 3 | 2.8 | 3.3 | 2.8 | 2.7 | 3 | 3.2 | 2.4 | 1.8 | 3.8 | 2.3 | 2.7 |
| **102** | **W 899** | 5 | 3.6 | 3.6 | 4.8 | 4.6 | 3.4 | 2.8 | 4 | 5.6 | 5.6 | 3.6 | 3.6 | 3.6 | 2.8 | 3 | 4.1 | 4 | 3 | 2.6 | 4.5 | 2.8 | 3 |
| **103** | **B 39** | 0 | 0 | 0 | 0.8 | 0 | 0 | 0 | 0 | 1 | 0 | 0 | 0 | 1 | 1 | 0 | 2.5 | 3 | 0.8 | 3 | 4.7 | 0 | 0 |
| **104** | **B 40** | 0 | 0 | 0 | 0.9 | 0 | 0 | 0 | 0 | 0 | 0 | 0 | 0 | 1 | 0.9 | 0 | 2.5 | 3.9 | 0 | 3.2 | 4.6 | 0 | 0 |
| **105** | **B 48** | 0 | 0 | 0 | 0.9 | 0 | 0 | 0 | 0 | 0 | 0 | 0 | 0 | 1 | 0 | 0 | 2.2 | 3.1 | 0.7 | 2.5 | 4.1 | 0 | 0 |
| **106** | **B 46** | 0 | 0 | 0 | 1 | 0 | 0 | 0 | 0 | 0 | 0 | 0 | 0 | 1 | 0 | 0 | 2.5 | 3.6 | 0.8 | 3.2 | 4.2 | 0 | 0 |
| **107** | **B 50** | 0 | 0 | 0 | 1.1 | 0 | 0 | 0 | 0 | 0.9 | 0 | 0 | 0 | 1 | 0 | 0 | 2.6 | 3.6 | 0.7 | 2.5 | 4.5 | 0 | 0 |
| **108** | **B 72** | 0 | 0 | 0 | 0.7 | 0 | 0 | 0 | 0 | 0 | 0 | 0 | 0 | 1 | 0 | 0 | 2 | 3.3 | 0 | 3 | 4 | 0 | 0 |
| **109** | **B 951** | 0 | 0 | 0 | 0.8 | 0 | 0 | 0 | 0 | 0 | 0 | 0 | 0 | 0.7 | 0.8 | 0 | 2.3 | 3.3 | 0.8 | 2.1 | 4.1 | 0 | 0 |
| **110** | **B 866** | 0 | 0 | 0 | 0.8 | 0 | 0 | 0 | 0 | 0 | 0 | 0 | 0 | 0 | 0 | 0 | 2.3 | 3.2 | 0.8 | 2.6 | 4 | 0 | 0 |
| **111** | **B 3** | 0 | 0 | 1 | 0 | 0 | 0 | 1 | 0 | 1 | 0 | 0 | 0 | 0.6 | 0 | 0 | 2.8 | 4 | 1.2 | 3 | 4.4 | 0 | 1.2 |
| **112** | **B 4** | 0 | 0 | 0 | 0.9 | 0 | 0 | 0 | 0 | 0 | 0 | 0 | 0 | 0.9 | 0.9 | 0 | 2.5 | 3.1 | 0 | 3 | 4.2 | 0 | 0 |
| **113** | **B 776** | 0 | 0 | 0 | 1.3 | 0 | 0 | 0 | 0 | 1 | 0 | 0.9 | 1 | 1 | 0 | 0 | 2.3 | 2.9 | 0.8 | 3.1 | 4 | 0 | 0 |
| **114** | **B 783** | 0 | 0 | 0 | 1 | 0 | 0 | 0 | 0 | 0 | 0 | 0 | 0 | 0.9 | 0.8 | 0 | 2.7 | 3.1 | 0.8 | 2.9 | 4 | 0 | 0 |
| **115** | **B 97** | 0 | 0 | 0 | 0.9 | 0 | 0 | 0 | 0 | 0 | 0 | 0 | 0 | 1 | 0 | 0 | 2.5 | 3.2 | 0.9 | 2.7 | 4 | 0 | 0 |
| **116** | **B 15** | 0 | 0 | 0 | 0.9 | 0 | 0 | 0 | 0 | 0 | 0 | 0.8 | 0.9 | 1 | 0 | 0 | 2.6 | 3.5 | 0.8 | 2.5 | 4.5 | 0 | 0 |
| **117** | **B 21** | 0 | 0 | 0 | 0 | 0 | 0 | 0 | 0 | 0 | 0 | 0 | 0 | 1.2 | 0.9 | 0 | 3 | 4.2 | 0.8 | 3.5 | 5.4 | 0 | 0 |
| **118** | **B 789** | 0 | 0 | 0 | 0.9 | 0 | 0 | 0 | 0 | 0.9 | 0 | 0.6 | 0.9 | 0.7 | 0 | 0 | 2.5 | 2.3 | 0.9 | 2 | 4 | 0 | 0 |
| **119** | **B 787** | 0 | 0 | 0 | 1 | 0 | 0 | 0 | 0 | 0.8 | 0 | 0 | 0 | 0.9 | 0 | 0 | 2.3 | 2.7 | 1.3 | 2.5 | 4 | 0 | 0 |
| **120** | **B 23** | 0 | 0 | 0 | 1.2 | 0 | 0 | 0 | 0 | 0 | 0 | 0 | 0 | 0.7 | 0.7 | 0 | 2.2 | 3.8 | 2.5 | 3.1 | 4.2 | 0 | 0.7 |
| **121** | **B 20** | 1 | 0 | 1 | 1.2 | 0 | 0 | 1 | 1 | 1.1 | 1.2 | 0.6 | 0.6 | 0.7 | 0.8 | 0 | 2.5 | 3.1 | 2.4 | 3 | 3.8 | 0.6 | 0.8 |

**Supplementary Table 1. Continued**

| **No.** | **Isolate code** | **P** | **OX** | **FOX** | **AMC** | **SAM** | **FEP** | **CXM** | **CEP** | **IPM** | **E** | **AZM** | **DA** | **C** | **AMK** | **CN** | **RA** | **SXT** | **DO** | **NF** | **LZ** | **NOR** | **CIP** |
| --- | --- | --- | --- | --- | --- | --- | --- | --- | --- | --- | --- | --- | --- | --- | --- | --- | --- | --- | --- | --- | --- | --- | --- |
| **122** | **B 14** | 1.4 | 0 | 0.9 | 1 | 0 | 0 | 1 | 1.4 | 1.2 | 1 | 0.8 | 0.8 | 3.1 | 0.9 | 0 | 2.5 | 3.5 | 0.6 | 2.9 | 4 | 0.6 | 0.8 |
| **123** | **B 868** | 0 | 0 | 0 | 1 | 2 | 0 | 0 | 1.4 | 1.3 | 0 | 0 | 0 | 0 | 0 | 0 | 2.3 | 3.2 | 0.8 | 2.6 | 4 | 0 | 0 |
| **124** | **B 864** | 0 | 0 | 0.9 | 1.2 | 0.9 | 0 | 0 | 2.4 | 3.8 | 0 | 0 | 0 | 1.2 | 0.6 | 0 | 2.3 | 3.5 | 1 | 3.3 | 5.6 | 0 | 0 |
| **125** | **B 856** | 0 | 0 | 0 | 1 | 0 | 0 | 0 | 0 | 0 | 4.2 | 4.4 | 4 | 1.1 | 1.1 | 0 | 2.2 | 3.9 | 0.9 | 3.4 | 4.2 | 0 | 0 |
| **126** | **B 950** | 0 | 0 | 0 | 1 | 0 | 0 | 0 | 0 | 1.2 | 4 | 3.5 | 3.7 | 1.1 | 0 | 0 | 1.9 | 3.6 | 1.2 | 2.6 | 4.4 | 0 | 0 |
| **127** | **B 774** | 0 | 0 | 0 | 1 | 0.9 | 0 | 0 | 0 | 1.3 | 3.5 | 3.1 | 3.2 | 1 | 1 | 0 | 2 | 2.4 | 1 | 2.6 | 4 | 0 | 0 |
| **128** | **B 975** | 1 | 0 | 1.5 | 1.7 | 1 | 0 | 0 | 2.7 | 1.3 | 1 | 1.5 | 3 | 3.2 | 0.9 | 0 | 3.7 | 0 | 1.2 | 2.8 | 4 | 0 | 0 |
| **129** | **B 786** | 0 | 0 | 0 | 0.9 | 0 | 0 | 0 | 0 | 0.9 | 3.1 | 3.2 | 3.2 | 0.9 | 0.9 | 0 | 2.3 | 2.2 | 1.2 | 2.5 | 4 | 0 | 0 |
| **130** | **B 791** | 0 | 0 | 0 | 1 | 0 | 0 | 0 | 0 | 1.2 | 3.6 | 3.3 | 3.6 | 0.9 | 0.9 | 0 | 2.3 | 2.5 | 1.3 | 2.7 | 4 | 0 | 0 |
| **131** | **B 974** | 0 | 0 | 0 | 1.2 | 0 | 0 | 0 | 0 | 0.9 | 4.6 | 4 | 4 | 2.8 | 0 | 0 | 2.8 | 4.5 | 0 | 2.8 | 5.3 | 0 | 0 |
| **132** | **B 856** | 0 | 0 | 0 | 1.2 | 0 | 0 | 0 | 0 | 0 | 4.7 | 4.6 | 4.6 | 2.9 | 0 | 0 | 2.9 | 4.1 | 0 | 3.1 | 5.4 | 0 | 0 |
| **133** | **B 956** | 0 | 0 | 0 | 1.4 | 0 | 0 | 0 | 0 | 0 | 4.1 | 3.6 | 4 | 3 | 0 | 0 | 2.4 | 3.8 | 0.8 | 3 | 4.7 | 0 | 0 |
| **134** | **B 954** | 1.6 | 0 | 0 | 0.9 | 1.3 | 0 | 0 | 0 | 3.4 | 5 | 4.8 | 5 | 1.4 | 0 | 0.6 | 3.2 | 4.7 | 1.1 | 3.3 | 5.4 | 0 | 0 |
| **135** | **B 14** | 1.4 | 0 | 1.5 | 1.4 | 0.9 | 1.3 | 0 | 1.9 | 1.2 | 3.6 | 3.4 | 3.5 | 1.2 | 2.7 | 2.9 | 3.8 | 2.7 | 1.2 | 2.8 | 4.3 | 1.1 | 1 |
| **136** | **B 865** | 0 | 0 | 2 | 1.2 | 0 | 3.4 | 1.8 | 2.5 | 1.2 | 0 | 0 | 0 | 0.9 | 2.8 | 2.8 | 2.3 | 4 | 3 | 3.4 | 4.3 | 1.6 | 1.6 |
| **137** | **B 760** | 1 | 0 | 1.8 | 1.3 | 1.2 | 0.8 | 0 | 1.6 | 4.2 | 1.2 | 1 | 1.9 | 2.7 | 2.5 | 2.7 | 2.5 | 0 | 3.5 | 2.5 | 4 | 2.7 | 3 |
| **138** | **B 31** | 1.1 | 0 | 2 | 2.1 | 1.6 | 1.7 | 0 | 1.9 | 1.3 | 0 | 0 | 3.2 | 2.8 | 2 | 2.9 | 3.6 | 3.6 | 3 | 2.5 | 4 | 1 | 0.9 |
| **139** | **B 84** | 1.3 | 1.5 | 2.1 | 2.4 | 1.8 | 1.7 | 0 | 1.7 | 3.9 | 0 | 0 | 3.3 | 2.7 | 1.4 | 1.2 | 3.2 | 3.4 | 1.1 | 2.4 | 3.7 | 0.9 | 1 |
| **140** | **B 26** | 1.3 | 0 | 1.7 | 2.4 | 2 | 1.6 | 0 | 1.8 | 3.7 | 0 | 0 | 3.4 | 1.2 | 2.5 | 1.5 | 3.7 | 3.6 | 1 | 2.4 | 3.7 | 3.3 | 3.1 |
| **141** | **B 767** | 2.7 | 1.2 | 2.3 | 2.8 | 2.4 | 2 | 0 | 2.2 | 4 | 0 | 0 | 0 | 1.2 | 2.4 | 2.4 | 3.3 | 0 | 1 | 2.4 | 4 | 1.5 | 0 |
| **142** | **B 764** | 1 | 0 | 1.3 | 1.8 | 1 | 0 | 0 | 1.8 | 3.5 | 3.3 | 3.4 | 3.4 | 3.2 | 2.6 | 3 | 3.8 | 4 | 1.4 | 2.6 | 4 | 3.2 | 3.3 |
| **143** | **B 771** | 3 | 1.4 | 5 | 2.5 | 2 | 0 | 5 | 0 | 4 | 3.5 | 3.2 | 3.6 | 0.9 | 1.4 | 0.8 | 1.3 | 5 | 1.8 | 2.4 | 4 | 0 | 0 |
| **144** | **B 969** | 1.3 | 0 | 1.4 | 1.8 | 1.8 | 1.7 | 0 | 1.7 | 3.1 | 3.3 | 3 | 3.2 | 1.1 | 2.3 | 2.3 | 3.4 | 2.8 | 1.2 | 2.5 | 4 | 2.6 | 2.8 |
| **145** | **B 91** | 1.5 | 0 | 2 | 2.2 | 1.7 | 1.8 | 0 | 1.8 | 3.8 | 3 | 3.1 | 3.6 | 3.2 | 1.3 | 1.2 | 3.6 | 3.5 | 0.9 | 2.6 | 3.9 | 3.2 | 3.2 |
| **146** | **B 86** | 1 | 0 | 1.6 | 2 | 1.8 | 1.4 | 0 | 2.6 | 3.1 | 2.9 | 3 | 3 | 1.1 | 2.3 | 2.1 | 3.3 | 2.5 | 0.9 | 2.2 | 4 | 2.6 | 2.8 |
| **147** | **B 94** | 0.9 | 0 | 1.6 | 2 | 0.9 | 0 | 0 | 1.7 | 3.2 | 3.3 | 3 | 3.3 | 3 | 2.4 | 2.5 | 3.5 | 2.5 | 2.6 | 2.3 | 4 | 2.7 | 2.6 |
| **148** | **B 964** | 2.3 | 0 | 1.5 | 3.6 | 2.5 | 0.8 | 0 | 2.5 | 2.7 | 2.4 | 2 | 0 | 2.5 | 1.5 | 1.5 | 2.3 | 3.7 | 2.8 | 2.4 | 3.8 | 1.7 | 2.6 |
| **149** | **B 658** | 1 | 0 | 1.9 | 2.5 | 1.8 | 1.8 | 0.8 | 2 | 3.6 | 3.4 | 3.2 | 2.9 | 2.7 | 1.6 | 0.8 | 3.6 | 3.3 | 2.6 | 2.1 | 3.7 | 2.8 | 3 |
| **150** | **B 2** | 1.6 | 0 | 2 | 2.3 | 2.2 | 1.7 | 0 | 2 | 4.2 | 3.4 | 3.3 | 3.3 | 3.1 | 2.3 | 1.5 | 3.8 | 3.6 | 1.2 | 2.3 | 4 | 3 | 3 |
| **151** | **B 38** | 1.8 | 0 | 2 | 2.5 | 2.3 | 1.8 | 0 | 1.9 | 3.9 | 3.2 | 3.3 | 3.4 | 3.4 | 2.7 | 1.6 | 3.7 | 3.7 | 0.9 | 2.7 | 3.8 | 2.7 | 2.6 |
| **152** | **B 71** | 1.6 | 0 | 2.1 | 2.3 | 1.5 | 2.8 | 1.3 | 2.7 | 6.2 | 3.6 | 3.6 | 3.2 | 3.5 | 1.1 | 0.6 | 4 | 3 | 2 | 2.8 | 4.2 | 3.2 | 4.2 |
| **153** | **B 187** | 1 | 0 | 1.6 | 2.3 | 1.9 | 1.7 | 0 | 1.7 | 3.3 | 3 | 3 | 2.7 | 2.8 | 2 | 2.1 | 3.5 | 3.5 | 1.7 | 2.1 | 3.7 | 2.3 | 2.4 |
| **154** | **B 701** | 1.1 | 0 | 1.5 | 2 | 1.3 | 1.5 | 0 | 1.9 | 3.5 | 3 | 2.7 | 3 | 2.6 | 2.2 | 2.2 | 3.4 | 1.9 | 2.3 | 2.1 | 3.5 | 2.4 | 2.5 |
| **155** | **B 858** | 0.9 | 0 | 2 | 2.2 | 1.5 | 1.8 | 0 | 2 | 4.4 | 3.8 | 4 | 3.6 | 3.4 | 3.1 | 3.1 | 3.7 | 2.5 | 3 | 2.8 | 4 | 3.2 | 3.6 |
| **156** | **B 971** | 2 | 1.3 | 2.1 | 2.4 | 2.2 | 2 | 0 | 2.3 | 3.6 | 3.6 | 3.6 | 3.7 | 3.2 | 2.4 | 2.2 | 4 | 3.3 | 1.2 | 2.3 | 3.7 | 2.7 | 2.1 |
| **157** | **B 27** | 1.5 | 0 | 1.9 | 2.1 | 1.5 | 2 | 0.9 | 2 | 4.2 | 3.4 | 3.3 | 3.3 | 2.9 | 2.5 | 1.3 | 3.3 | 2.8 | 3.2 | 2 | 3.6 | 2.7 | 3 |
| **158** | **B 70** | 1.6 | 0 | 1.5 | 2.1 | 1.5 | 1.9 | 0 | 1.9 | 3.1 | 3.4 | 3.3 | 3.2 | 3.4 | 2.2 | 2.8 | 3.8 | 3.9 | 2.6 | 2.5 | 4 | 2.6 | 3 |
| **159** | **B 777** | 4.6 | 2.6 | 3 | 4.6 | 3.8 | 2.3 | 1 | 3.4 | 5.3 | 0 | 0 | 1.5 | 1.3 | 1.2 | 0.8 | 3.5 | 2.5 | 1.5 | 2.5 | 4 | 1.1 | 2 |
| **160** | **B 660** | 2.4 | 0 | 2.3 | 3.4 | 2.2 | 2.7 | 1.1 | 2.6 | 5.2 | 4.4 | 4.2 | 4 | 4.4 | 2.7 | 3.2 | 4.8 | 4.5 | 3 | 2.7 | 4.5 | 4 | 4 |
| **161** | **B 44** | 3 | 2.3 | 3.4 | 3.6 | 3 | 1.4 | 1.2 | 2.9 | 4 | 0 | 0 | 3.8 | 3.4 | 3.4 | 3.4 | 4 | 3.8 | 1.2 | 3.4 | 4.4 | 3.6 | 3.6 |

**Supplementary Table 1. Continued**

| **No.** | **Isolate code** | **P** | **OX** | **FOX** | **AMC** | **SAM** | **FEP** | **CXM** | **CEP** | **IPM** | **E** | **AZM** | **DA** | **C** | **AMK** | **CN** | **RA** | **SXT** | **DO** | **NF** | **LZ** | **NOR** | **CIP** |
| --- | --- | --- | --- | --- | --- | --- | --- | --- | --- | --- | --- | --- | --- | --- | --- | --- | --- | --- | --- | --- | --- | --- | --- |
| **162** | **B 89** | 1.7 | 0 | 2.4 | 2.5 | 2.2 | 2 | 0.9 | 2 | 4 | 3.3 | 3.3 | 3 | 3.2 | 2.7 | 1.6 | 3.9 | 3.7 | 1.7 | 2.8 | 3.8 | 3.1 | 3.2 |
| **163** | **B 762** | 1.3 | 1.2 | 1.8 | 2.1 | 1.6 | 2 | 0 | 2 | 4.4 | 2.8 | 3 | 2.7 | 2.5 | 2.2 | 1.4 | 3.1 | 2.5 | 1.4 | 2.2 | 4 | 2.7 | 3 |
| **164** | **B 769** | 1.2 | 1.2 | 1.8 | 2.3 | 1.7 | 1.8 | 0 | 1.9 | 5 | 3.2 | 3.3 | 3.3 | 2.8 | 2.4 | 1.4 | 3.7 | 2.5 | 1.5 | 2.3 | 4 | 3 | 3.2 |
| **165** | **B 37** | 1.9 | 1.3 | 2.2 | 2.7 | 2 | 2.1 | 0 | 2.2 | 4.1 | 3.3 | 3 | 2.7 | 2.6 | 1.9 | 0.9 | 3.8 | 3.5 | 1.3 | 2.3 | 3.8 | 2.7 | 2.9 |
| **166** | **B 342** | 2.6 | 1.7 | 2.6 | 3.4 | 2.6 | 2.3 | 0 | 2.8 | 4.8 | 4.2 | 3.9 | 3.5 | 3.8 | 2.4 | 3.1 | 4.3 | 4.1 | 1.4 | 2.7 | 4.4 | 3.2 | 3.3 |
| **167** | **B 933** | 2 | 1.4 | 2.2 | 2.6 | 2.1 | 2.1 | 2 | 2.4 | 4.4 | 3.6 | 3.2 | 3.4 | 3 | 2.2 | 1.3 | 3.8 | 3.6 | 0.8 | 2.2 | 4.2 | 3.2 | 3.4 |
| **168** | **B 955** | 2.4 | 1.4 | 2.4 | 2.9 | 2.6 | 2.2 | 0 | 2.3 | 4.4 | 4 | 3.6 | 3.2 | 2.8 | 2.5 | 3.1 | 3.7 | 3.3 | 1.5 | 2.1 | 4 | 2.7 | 3 |
| **169** | **B 973** | 2.1 | 2 | 3 | 3 | 2.4 | 2.8 | 1.1 | 2.7 | 5.6 | 3.6 | 3.4 | 3.4 | 3.4 | 2.7 | 2.8 | 3.7 | 3.8 | 3.4 | 2.8 | 4.3 | 3 | 3.2 |
| **170** | **B 16** | 1.9 | 2.1 | 3.2 | 2.9 | 2.3 | 2.4 | 1.4 | 2.5 | 4 | 3.5 | 3.3 | 3.4 | 3.4 | 2.5 | 2.7 | 3.7 | 4 | 2.5 | 2.6 | 3.8 | 2.8 | 2.8 |
| **171** | **B 24** | 2.8 | 2 | 3.6 | 3.4 | 2.8 | 3.2 | 1 | 2.8 | 4 | 4 | 5.4 | 4 | 3.4 | 3.2 | 3 | 4 | 4.2 | 2.6 | 2.8 | 4.4 | 3 | 3.6 |
| **172** | **B 770** | 1.6 | 1.5 | 2 | 2.5 | 2 | 2.2 | 0 | 2.1 | 4.7 | 3.1 | 3.2 | 3.3 | 2.9 | 2.5 | 2.6 | 3.5 | 2.7 | 3.4 | 2.2 | 4 | 3 | 3 |
| **173** | **B 765** | 1.7 | 1.7 | 2.4 | 2.5 | 2.1 | 2.2 | 0.7 | 2.2 | 5.6 | 3.3 | 3.4 | 3 | 2.8 | 2.5 | 2.7 | 3.7 | 3.4 | 2.6 | 2.6 | 4 | 2.8 | 2.8 |
| **174** | **B 78** | 1.4 | 2.5 | 2.4 | 3 | 2.1 | 2.4 | 3.4 | 2.5 | 4.8 | 1.5 | 1.6 | 3.1 | 3 | 2.2 | 2.2 | 3.8 | 3 | 3 | 2.8 | 4.6 | 2.6 | 2.8 |
| **175** | **B 29** | 2.6 | 1.4 | 3 | 3 | 3.2 | 3.4 | 3.8 | 2.6 | 5 | 3.5 | 3.4 | 3.4 | 3.4 | 3.6 | 1.6 | 4 | 3.8 | 3 | 3.6 | 4.4 | 3.4 | 3.6 |
| **176** | **B 918** | 3 | S | 3.4 | S | S | 3 | 0 | 3.6 | 4.5 | S | 4 | 3.8 | 3.4 | 3.6 | 3.4 | 5 | 3.2 | 2.8 | 3.2 | 4.4 | 3.2 | 3 |
| **177** | **U 426** | 0 | 1 | 0 | 1.1 | 0.9 | 0 | 0 | 0 | 0 | 0 | 0 | 0 | 1 | 0 | 0 | 3 | 3.3 | 0.9 | 3.1 | 4.7 | 0 | 0 |
| **178** | **U 418** | 0 | 0 | 0 | 0.8 | 0 | 0 | 0 | 0 | 0 | 0 | 0 | 0 | 1 | 0.8 | 0 | 2.1 | 3.5 | 3.2 | 2.3 | 4.5 | 0 | 0.6 |
| **179** | **U 399** | 0 | 0 | 0 | 1 | 0 | 0 | 0 | 0 | 0.7 | 3.7 | 3.4 | 3.6 | 3 | 0 | 0 | 2 | 3.3 | 3 | 2.7 | 4.2 | 0 | 0 |
| **180** | **U 674** | 0 | 0 | 1.5 | 1.7 | 2.1 | 0 | 0 | 2.3 | 3.4 | 3 | 2.9 | 3 | 3.1 | 2.3 | 2.6 | 3.6 | 2.4 | 2.6 | 2.2 | 4 | 2.5 | 2.4 |
| **181** | **U 16** | 1.5 | 0.9 | 2 | 2.4 | 2.1 | 1.8 | 0 | 2.3 | 4.2 | 3.2 | 3 | 2.6 | 2.5 | 1.7 | 2.9 | 3.6 | 3.4 | 1.2 | 2.2 | 3.7 | 2.6 | 2.6 |
| **182** | **U 419** | 1.5 | 0.8 | 2 | 2.4 | 1.8 | 2 | 1.7 | 2.6 | 3.3 | 3.5 | 3.6 | 3.6 | 2.8 | 2.1 | 2.5 | 3.4 | 2.3 | 2.7 | 2 | 3.7 | 0.9 | 0.9 |
| **183** | **U 22** | 1.3 | 0 | 1.8 | 2.2 | 2 | 1.6 | 0 | 2 | 4 | 3.6 | 3.6 | 3.6 | 3.4 | 2.8 | 3 | 3.3 | 3.1 | 2.6 | 2.8 | 4 | 3.2 | 3 |
| **184** | **U 427** | 1.6 | 0 | 1.7 | 2.3 | 2 | 1.7 | 0 | 2 | 3.3 | 3 | 3.1 | 3.1 | 2.7 | 2.2 | 2.1 | 3.3 | 3.3 | 1.5 | 2.2 | 3.6 | 2.4 | 2.5 |
| **185** | **U 445** | 1.5 | 0.9 | 2.1 | 2.3 | 1.9 | 1.8 | 1.9 | 1.9 | 3.5 | 3 | 3 | 3.2 | 2.8 | 2.1 | 2.8 | 3.6 | 3.6 | 1.5 | 2.2 | 3.9 | 3 | 3 |
| **186** | **U 141** | 2 | 1.7 | 3 | 2.8 | 2.2 | 2.2 | 0.9 | 2.4 | 5 | 1.7 | 1.6 | 0 | 3.2 | 2.4 | 2.4 | 1.9 | 3.4 | 2.8 | 2.6 | 3.8 | 1.1 | 1.8 |
| **187** | **U 314** | 2.1 | 1 | 2.3 | 2.3 | 2.1 | 2 | 0 | 2.1 | 3.6 | 3.3 | 2.9 | 2.7 | 2.6 | 1.8 | 2.8 | 3.3 | 3.6 | 1.4 | 2.1 | 4 | 2.6 | 2.9 |
| **188** | **U 202** | 2.4 | 1.4 | 2.2 | 2.8 | 2.5 | 2.1 | 0 | 2.2 | 4.2 | 3.6 | 3.2 | 3.6 | 1.3 | 2.3 | 2.4 | 3.4 | 3.5 | 1.1 | 2.3 | 4 | 2.3 | 2.9 |
| **189** | **U 944** | 2.2 | 1.6 | 2.2 | 2.6 | 2.4 | 2.2 | 2.2 | 2.4 | 4.4 | 3.8 | 3.4 | 3.6 | 3.4 | 2.4 | 1.3 | 4 | 3.8 | 0.9 | 2.6 | 4.5 | 3.4 | 3.4 |
| **190** | **U 211** | 2 | 1.6 | 2.8 | 2.6 | 2 | 2.4 | 0.9 | 2.2 | 4.8 | 2.9 | 3 | 2.8 | 3.4 | 2.1 | 2.5 | 3.8 | 3.7 | 1.6 | 2.4 | 4 | 2.5 | 2.6 |
| **191** | **U 945** | 2 | 1.7 | 3 | 2.7 | 1.8 | 2.4 | 2 | 2.3 | 4 | 3.6 | 3.2 | 2.6 | 2.5 | 2.5 | 2.4 | 3.8 | 3.2 | 3.4 | 2.1 | 4 | 2.6 | 2.5 |
| **192** | **U 430** | 2 | 2.3 | 3.4 | 2.8 | 2.2 | 3 | 3.4 | 2.5 | 5.4 | 3.6 | 3.2 | 3.2 | 3 | 2.5 | 2.4 | 3.5 | 3.7 | 2.8 | 2.3 | 4.4 | 2.1 | 3 |
| **193** | **U 431** | 1.8 | 1.7 | 2.8 | 2.5 | 2.2 | 2.2 | 2.6 | 2.4 | 4 | 3.1 | 3.3 | 3.5 | 3.1 | 2.5 | 2.5 | 3.3 | 3.2 | 3 | 2.4 | 3.6 | 2.7 | 2.5 |
| **194** | **U 432** | 2 | 1.8 | 3.6 | 3 | 2.4 | 2.2 | 3 | 2.6 | 4.8 | 3.6 | 3.6 | 3.4 | 3.3 | 2.5 | 2.6 | 4.3 | 4.4 | 3 | 2.5 | 4.5 | 2.4 | 3 |
| **195** | **U 17** | 2.6 | 2.5 | 2.5 | 3.4 | 2.6 | 2.4 | 2.6 | 2.7 | 4.5 | 2.8 | 2.1 | 2.8 | 1.2 | 2 | 2.1 | 3.6 | 1.3 | 2.1 | 2.8 | 4.1 | 2.6 | 2.8 |
| **196** | **BL 14** | 0 | 0 | 0 | 1 | 0 | 0 | 0 | 0 | 0 | 4.2 | 4 | 4 | 3.6 | 0 | 0 | 2 | 3.8 | 0.8 | 3.2 | 4.3 | 0 | 0 |
| **197** | **BL 7** | 1.3 | 0 | 2.1 | 2.5 | 2.4 | 1.3 | 0 | 2.4 | 3.7 | 0 | 0 | 3.6 | 3 | 2.6 | 1.4 | 4 | 4.2 | 2.8 | 2.6 | 4.3 | 3.2 | 3 |
| **198** | **BL 11** | 0.9 | 0 | 1.6 | 1.7 | 2 | 1.3 | 0 | 1.9 | 3.5 | 3.7 | 3.6 | 3.4 | 1.3 | 2.6 | 3 | 3.9 | 3.3 | 1.4 | 2.6 | 4.1 | 3 | 3.2 |
| **199** | **BL 5** | 1.1 | 0 | 1.5 | 2 | 2.4 | 2 | 0 | 2.2 | 3.4 | 3 | 3.2 | 3 | 3 | 2.3 | 2.3 | 3.7 | 3.5 | 2.5 | 2.4 | 4.2 | 2.6 | 2.7 |
| **200** | **BL 12** | 1.4 | 1.4 | 2.2 | 2.6 | 2 | 1.9 | 1.8 | 2 | 4 | 3.2 | 2.9 | 2.8 | 2.8 | 1.2 | 0.7 | 3.5 | 3.6 | 1.3 | 2.1 | 3.7 | 2.6 | 2.8 |
| **201** | **S 437** | 0 | 0 | 0 | 1.1 | 1.2 | 0 | 0 | 0 | 0.9 | 0 | 0 | 0 | 0.9 | 0 | 0 | 2.3 | 3.6 | 0 | 2.8 | 4.6 | 0 | 0 |

**Supplementary Table 1.** **Continued**

| **No.** | **Isolate code** | **P** | **OX** | **FOX** | **AMC** | **SAM** | **FEP** | **CXM** | **CEP** | **IPM** | **E** | **AZM** | **DA** | **C** | **AMK** | **CN** | **RA** | **SXT** | **DO** | **NF** | **LZ** | **NOR** | **CIP** |
| --- | --- | --- | --- | --- | --- | --- | --- | --- | --- | --- | --- | --- | --- | --- | --- | --- | --- | --- | --- | --- | --- | --- | --- |
| **202** | **S 417** | 0 | 0 | 0 | 1.6 | 0 | 0 | 1.5 | 0 | 1.3 | 3.4 | 3.1 | 3.1 | 0.9 | 0.9 | 0 | 0.8 | 0 | 0 | 1.8 | 4.2 | 0 | 0 |
| **203** | **S 445** | 1.1 | 0 | 1.8 | 2.5 | 2.1 | 1.2 | 0 | 2.7 | 3.7 | 3.2 | 3 | 3 | 2.6 | 2.2 | 2.3 | 3.6 | 3.2 | 2.8 | 2.3 | 4 | 2.5 | 2.6 |
| **204** | **S 441** | 2.4 | 1.6 | 2.2 | 2.2 | 2 | 1.8 | 0 | 2 | 4.4 | 2.8 | 2.6 | 2.8 | 2.6 | 2 | 2.9 | 4.4 | 3.4 | 1 | 2 | 3.6 | 2.2 | 2.5 |
| **205** | **S 442** | 1.6 | 0 | 2.7 | 2.3 | 2.1 | 2.2 | 2.2 | 2 | 3.7 | 3.5 | 3.5 | 3.6 | 3.5 | 2.3 | 2.9 | 4 | 4 | 1.2 | 2.5 | 4.3 | 2.6 | 3 |
| **206** | **E 189** | 0 | 0 | 0 | 1.1 | 0 | 0 | 0 | 0 | 0 | 0 | 0 | 0 | 0.9 | 1 | 0 | 0.6 | 3.3 | 1 | 2.5 | 4 | 0 | 0 |
| **207** | **E 444** | 1 | 0 | 1.2 | 2.6 | 1.6 | 1.8 | 0.8 | 2 | 3.6 | 3.3 | 3.1 | 3.2 | 1 | 2.5 | 2.4 | 3.6 | 1 | 1.1 | 2.5 | 3.7 | 0.7 | 0.8 |
| **208** | **E 302** | 1.6 | 1.1 | 2 | 2.2 | 1.7 | 2 | 0.9 | 2 | 4.1 | 3.1 | 3 | 2.6 | 2.9 | 1.2 | 1 | 3.8 | 3.6 | 1.5 | 2 | 4 | 2.8 | 3 |
| **209** | **E 434** | 1.4 | 0 | 1.8 | 2.2 | 1.7 | 1.6 | 1.3 | 2.3 | 3.7 | 3.6 | 3.4 | 3.4 | 3 | 2.3 | 2.7 | 4 | 3.5 | 2.5 | 2.6 | 3.8 | 2.5 | 2.6 |

**No., the isolate number, W, isolate source is wound; B, isolate source is burn; U, isolate source is urine; BL, isolate source is blood; S, isolate source is sputum; E, isolate source is Endotracheal aspirate; P, penicillin G; OX, oxacillin; FOX, cefoxitin; AMC, amoxicillin / clavulanic acid; SAM, ampicillin / sulbactam; FEP, cefepime; CXM, ceforuxime; CFP, cefoperazone; IMP, imipenem; E, erythromycin; AZM, azithromycin; DA, clindamycin; C, chloramphenicol; AMK, amikacin; CN, gentamicin; RA, rifampin; SXT, sulphamethoxazole / trimethoprim; DO, doxycycline; NF, nitrofurantoin; LZ, linezolid; NOR, norfloxacin; CIP, ciprofloxacin.**
