# Supplementary material for: Promising FDA-approved drugs with efflux pump inhibitory activities against clinical isolates of Staphylococcus aureus
Source: PLoS One. 2022 Jul 29;17(7):e0272417. doi: 10.1371/journal.pone.0272417 (PMC9337675; doi:10.1371/journal.pone.0272417)
Supplement: S2 Table — (DOCX) [file pone.0272417.s002.docx]

**Supplementary Table 2.** **Zone diameter breakpoints of tested antibiotics according to CLSI (2012)**

| **Antibiotic** | **Zone diameter breakpoints**  **(Nearest whole cm)** | | |
| --- | --- | --- | --- |
|  | **Resistant**  **(R)** | **Intermediate (I)** | **Sensitive**  **(S)** |
| **Penicillin (P)** | ≤ 2.8 | - | ≥ 2.9 |
| **Oxacillin (OX)** | ≤ 1.0 | 1.1 - 1.2 | ≥ 1.3 |
| **Amoxicillin / Clavulanic acid (AMC)** | ≤ 1.9 | - | ≥ 2.0 |
| **Ampicillin / Sulbactam (SAM)** | ≤ 1.1 | 1.2 - 1.4 | ≥ 1.5 |
| **Cefoxitin (FOX)** | ≤ 2.1 | - | ≥ 2.2 |
| **Cefepime (FEP)** | ≤ 1.4 | 1.5 - 1.7 | ≥ 1.8 |
| **Ceforuxime (CXM)** | ≤ 1.4 | 1.5 - 1.7 | ≥ 1.8 |
| **Cefoperazone (CEP)** | ≤ 1.5 | 1.6 - 2.0 | ≥ 2.1 |
| **Imipenem (IMP)** | ≤ 1.3 | 1.4 - 1.5 | ≥ 1.6 |
| **Erythromycin (E)** | ≤ 1.3 | 1.4 - 2.2 | ≥ 2.3 |
| **Azithromycin (AZM)** | ≤ 1.3 | 1.4 - 1.7 | ≥ 1.8 |
| **Clindamycin (DA)** | ≤ 1.4 | 1.5 - 2.0 | ≥ 2.1 |
| **Chloramphenicol (C)** | ≤ 1.2 | 1.3 - 1.7 | ≥ 1.8 |
| **Gentamicin (CN)** | ≤ 1.2 | 1.3 - 1.4 | ≥ 1.5 |
| **Amikacin (AMK)** | ≤ 1.4 | 1.5 - 1.6 | ≥ 1.7 |
| **Rifampin (RA)** | ≤ 1.6 | 1.7 - 1.9 | ≥ 2.0 |
| **Sulphamethoxazole / Trimethoprim (SXT)** | ≤ 1.0 | 1.1 - 1.5 | ≥ 1.6 |
| **Doxycycline (DO)** | ≤ 1.2 | 1.3 - 1.5 | ≥ 1.6 |
| **Nitrofurantoin (NF)** | ≤ 1.4 | 1.5 - 1.6 | ≥ 1.7 |
| **Linezolid (LZ)** | ≤ 2.0 | - | ≥ 2.1 |
| **Norfloxacin (NOR)** | ≤ 1.2 | 1.3 - 1.6 | ≥ 1.7 |
| **Ciprofloxacin (CIP)** | ≤ 1.5 | 1.6 - 2.0 | ≥ 2.1 |
