# Supplementary material for: Promising FDA-approved drugs with efflux pump inhibitory activities against clinical isolates of Staphylococcus aureus
Source: PLoS One. 2022 Jul 29;17(7):e0272417. doi: 10.1371/journal.pone.0272417 (PMC9337675; doi:10.1371/journal.pone.0272417)
Supplement: S3 Table — R, resistant isolates; I, intermediate resistant isolate; S, sensitive isolates, No., number; %, percentage. (DOCX) [file pone.0272417.s003.docx]

**Supplementary Table 3.** **Resistance patterns in the total isolates (n=209)**

| Antibiotic | Resistance pattern in the total isolates (n=209) | | | | | |
| --- | --- | --- | --- | --- | --- | --- |
|  | **R** | | **I** | | **S** | |
|  | **No.** | **%** | **No.** | **%** | **No.** | **%** |
| Penicillin (P) | 197 | 94.3% | 0 | 0.0% | 12 | 5.7% |
| Cefuroxime (CXM) | 169 | 80.9% | 8 | 3.8% | 32 | 15.3% |
| Cefoxitin (FOX) | 134 | 64.1% | 0 | 0.0% | 75 | 35.9% |
| Oxacillin (OX) | 132 | 63.2% | 8 | 3.8% | 69 | 33.0% |
| Doxycycline (DO) | 82 | 39.2% | 30 | 14.4% | 97 | 46.4% |
| Gentamicin (CN) | 78 | 37.3% | 15 | 7.2% | 116 | 55.5% |
| Norfloxacin (NOR) | 75 | 35.9% | 3 | 1.4% | 131 | 62.7% |
| Amikacin (AMK) | 72 | 34.4% | 7 | 3.3% | 130 | 62.2% |
| Cefepime (FEP) | 70 | 33.5% | 21 | 10.0% | 118 | 56.5% |
| Ciprofloxacin (CIP) | 69 | 33.0% | 7 | 3.3% | 133 | 63.6% |
| Amoxicillin/Clavulanic acid (AMC) | 68 | 32.5% | 0 | 0.0% | 141 | 67.5% |
| Erythromycin (E) | 61 | 29.2% | 4 | 1.9% | 144 | 68.9% |
| Azithromycin (AZM) | 61 | 29.2% | 4 | 1.9% | 144 | 68.9% |
| Imipenem (IMP) | 56 | 26.8% | 0 | 0.0% | 153 | 73.2% |
| Chloramphenicol (C) | 55 | 26.3% | 6 | 2.9% | 148 | 70.8% |
| Ampicillin/Sulbactam (SAM) | 55 | 26.3% | 7 | 3.3% | 147 | 70.3% |
| Cefoperazone (CEP) | 55 | 26.3% | 53 | 25.4% | 101 | 48.3% |
| Clindamycin (DA) | 42 | 20.1% | 5 | 2.4% | 162 | 77.5% |
| Sulfamethoxazole/Trimethoprim (SXT) | 7 | 3.3% | 5 | 2.4% | 197 | 94.3% |
| Rifampin (RA) | 5 | 2.4% | 5 | 2.4% | 199 | 95.2% |
| Nitrofurantoin (NF) | 0 | 0.0% | 0 | 0.0% | 209 | 100.0% |
| Linezolid (LZ) | 0 | 0.0% | 0 | 0.0% | 209 | 100.0% |

**R, resistant isolates; I, intermediate resistant isolate; S, sensitive isolates, No., number; %, percentage.**
