# Supplementary material for: Promising FDA-approved drugs with efflux pump inhibitory activities against clinical isolates of Staphylococcus aureus
Source: PLoS One. 2022 Jul 29;17(7):e0272417. doi: 10.1371/journal.pone.0272417 (PMC9337675; doi:10.1371/journal.pone.0272417)
Supplement: S4 Table — W, wound is the isolate source; B, burn is the isolate source; U, urine is the isolate source; S, sputum is the isolate source; E, Endotracheal aspirate is the isolate source; P, penicillin G; OX, oxacillin; AMC, amoxicillin / clavulanic acid; SAM, ampicillin / sulbactam; FEP, cefepime; CXM, ceforuxime; CFP, cefoperazone; IMP, imipenem; E, erythromycin; AZM, azithromycin; DA, clindamycin; C, chloramphenicol; AMK, amikacin; CN, gentamicin; RA, rifampin; SXT, sulphamethoxazole / trimethoprim; DO, doxycycline; NOR, norfloxacin; CIP, ciprofloxacin. The antibiotics, mentioned in each row, are those to which each isolate was resistant. (DOCX) [file pone.0272417.s004.docx]

**Supplementary Table 4.** **The resistance profile and the source of the selected isolates (n=72)**

| **Resistance phenotype** | | | | | | | | | | | | | | | | | | | **No. of**  **isolates** | **Isolates source** |
| --- | --- | --- | --- | --- | --- | --- | --- | --- | --- | --- | --- | --- | --- | --- | --- | --- | --- | --- | --- | --- |
| P | OX | FOX | AMC | SAM | FEP | CXM | CEP | IPM | CIP | NOR | E | AZM | DA | CN | AMK | DO | C | RA | 1 | E |
| P | OX | FOX | AMC | SAM | FEP | CXM | CEP | IPM | CIP | NOR | E | AZM | DA | CN | AMK | DO | C |  | 20 | 16 B, 3 W, 1 U |
| P | OX | FOX | AMC | SAM | FEP | CEP | IPM | CIP | NOR | E | AZM | DA | CN | AMK | DO | C |  |  | 1 | W |
| P | OX | FOX | AMC | FEP | CXM | CEP | IPM | CIP | NOR | E | AZM | DA | CN | AMK | DO | C |  |  | 2 | W, S |
| P | OX | FOX | AMC | SAM | FEP | CXM | CEP | IPM | CIP | NOR | E | AZM | DA | CN | AMK | C |  |  | 3 | B |
| P | OX | FOX | AMC | SAM | FEP | CXM | CEP | IPM | CIP | NOR | E | AZM | DA | CN | AMK | DO |  |  | 4 | 1 B, 3 W |
| P | OX | FOX | AMC | SAM | FEP | CXM | CEP | IPM | CIP | NOR | E | AZM | DA | CN | AMK | C |  |  | 1 | U |
| P | OX | FOX | AMC | SAM | FEP | CEP | IPM | CIP | NOR | CN | AMK | DO | C | RA | SXT |  |  |  | 1 | S |
| P | OX | FOX | AMC | SAM | FEP | CXM | CIP | NOR | E | AZM | DA | CN | AMK | DO | C |  |  |  | 1 | B |
| P | OX | FOX | FEP | CXM | CEP | IPM | CIP | NOR | E | AZM | DA | CN | AMK | DO | C |  |  |  | 1 | B |
| P | OX | FOX | AMC | SAM | FEP | CXM | CEP | IPM | CIP | NOR | E | AZM | DA | CN | DO |  |  |  | 1 | W |
| P | OX | FOX | AMC | SAM | FEP | CXM | CEP | IPM | CIP | NOR | CN | AMK | DO | C | RA |  |  |  | 1 | W |
| P | OX | FOX | AMC | FEP | CEP | IPM | CIP | NOR | E | AZM | DA | CN | AMK | DO |  |  |  |  | 1 | W |
| P | OX | FOX | AMC | SAM | FEP | CXM | IPM | CIP | NOR | E | CN | AMK | DO | SXT |  |  |  |  | 1 | B |
| P | OX | FOX | AMC | SAM | FEP | CXM | CEP | IPM | CIP | NOR | CN | AMK | DO | C |  |  |  |  | 5 | 4 B, 1 W |
| P | OX | FOX | AMC | SAM | FEP | CXM | CEP | IPM | CIP | NOR | CN | AMK | DO | RA |  |  |  |  | 1 | W |
| P | OX | FOX | AMC | FEP | CXM | CEP | IPM | CIP | NOR | CN | AMK | DO | SXT |  |  |  |  |  | 1 | W |
| P | OX | FOX | AMC | SAM | FEP | CXM | CEP | IPM | CIP | NOR | CN | AMK | DO |  |  |  |  |  | 3 | B |
| P | OX | FOX | AMC | SAM | FEP | CXM | CEP | IPM | CIP | NOR | CN | AMK | C |  |  |  |  |  | 1 | B |
| P | OX | FOX | AMC | SAM | FEP | CXM | CEP | IPM | CIP | NOR | CN | AMK | DO |  |  |  |  |  | 1 | W |
| P | OX | FOX | AMC | SAM | FEP | CXM | CEP | IPM | CIP | NOR | CN | AMK |  |  |  |  |  |  | 1 | U |
| P | OX | FOX | AMC | SAM | FEP | CXM | IPM | CIP | NOR | DO | C |  |  |  |  |  |  |  | 1 | B |
| P | OX | FOX | AMC | FEP | CXM | CEP | IPM | CIP | NOR | CN | DO |  |  |  |  |  |  |  | 1 | W |
| P | OX | FOX | AMC | SAM | FEP | CXM | CEP | CIP | NOR | E | AZM |  |  |  |  |  |  |  | 1 | W |
| P | CXM | CIP | NOR | E | AZM | DA | CN | AMK | DO | C |  |  |  |  |  |  |  |  | 1 | W |
| P | OX | FOX | AMC | SAM | IPM | E | AZM | DA | C |  |  |  |  |  |  |  |  |  | 1 | B |
| P | FOX | CXM | CIP | NOR | E | AZM | CN | AMK | DO |  |  |  |  |  |  |  |  |  | 1 | B |
| P | CXM | CIP | E | AZM | DA | DO | C | SXT |  |  |  |  |  |  |  |  |  |  | 1 | B |
| P | OX | FOX | CXM | CIP | NOR | DO | C | SXT |  |  |  |  |  |  |  |  |  |  | 1 | E |
| P | OX | FOX | CXM | IPM | CIP | NOR | E | AZM |  |  |  |  |  |  |  |  |  |  | 1 | B |
| P | CXM | CIP | NOR | E | AZM | DA | DO | C |  |  |  |  |  |  |  |  |  |  | 1 | W |
| P | OX | FOX | CXM | E | AZM | DO | C |  |  |  |  |  |  |  |  |  |  |  | 1 | B |
| FEP | CEP | CIP | NOR | CN | AMK | C | RA |  |  |  |  |  |  |  |  |  |  |  | 1 | B |
| P | OX | FOX | AMC | CXM | DO | C |  |  |  |  |  |  |  |  |  |  |  |  | 1 | B |
| P | FOX | CXM | CIP | NOR | DO | C |  |  |  |  |  |  |  |  |  |  |  |  | 1 | W |
| P | OX | FOX | CIP | NOR |  |  |  |  |  |  |  |  |  |  |  |  |  |  | 3 | 2 W, 1 U |
| P | OX | FOX | CXM |  |  |  |  |  |  |  |  |  |  |  |  |  |  |  | 1 | W |
| P | OX | FOX |  |  |  |  |  |  |  |  |  |  |  |  |  |  |  |  | 2 | W |

**W, wound is the isolate source; B, burn is the isolate source; U, urine is the isolate source; S, sputum is the isolate source; E, Endotracheal aspirate is the isolate source; P, penicillin G; OX, oxacillin; AMC, amoxicillin / clavulanic acid; SAM, ampicillin / sulbactam; FEP, cefepime; CXM, ceforuxime; CFP, cefoperazone; IMP, imipenem; E, erythromycin; AZM, azithromycin; DA, clindamycin; C, chloramphenicol; AMK, amikacin; CN, gentamicin; RA, rifampin; SXT, sulphamethoxazole / trimethoprim; DO, doxycycline; NOR, norfloxacin; CIP, ciprofloxacin.** **The antibiotics, mentioned in each row, are those to which each isolate was resistant.**
