# Supplementary material for: Promising FDA-approved drugs with efflux pump inhibitory activities against clinical isolates of Staphylococcus aureus
Source: PLoS One. 2022 Jul 29;17(7):e0272417. doi: 10.1371/journal.pone.0272417 (PMC9337675; doi:10.1371/journal.pone.0272417)
Supplement: S5 Table — * The number shown in brackets beside each antibiotic represents the number of resistant isolates to that antibiotic. (DOCX) [file pone.0272417.s005.docx]

**Supplementary Table 5. Number of antibiotic resistant isolates that harbored the efflux genes from which the statistical analysis of the correlations was made**

| **Number of resistant isolates** | **Number of efflux genes present** | | | |
| --- | --- | --- | --- | --- |
|  | ***tet*K** | ***nor*A** | ***fex*A** | ***msr*A** |
| **Penicillin (71)*** | 57 | 66 | 22 | 5 |
| **Oxacillin (66)** | 52 | 61 | 20 | 4 |
| **Cefoxitin (68)** | 54 | 63 | 21 | 5 |
| **Imipenem (55)** | 48 | 55 | 18 | 2 |
| **Erythromycin (45)** | 38 | 44 | 17 | 5 |
| **Azithromycin (44)** | 37 | 43 | 17 | 4 |
| **Clindamycin (40)** | 36 | 40 | 17 | 0 |
| **Chloramphenicol (48)** | 40 | 45 | 23 | 1 |
| **Amikacin (54)** | 49 | 53 | 19 | 2 |
| **Gentamicin (56)** | 51 | 55 | 19 | 2 |
| **Doxycycline (56)** | 52 | 54 | 19 | 3 |
| **Norfloxacin (65)** | 55 | 64 | 22 | 4 |
| **Ciprofloxacin (66)** | 56 | 65 | 22 | 4 |
| **Rifampin (5)** | 3 | 4 | 2 | 0 |
| **Sulfamethoxazole/Trimethoprim (5)** | 2 | 5 | 1 | 1 |

*** The number shown in brackets beside each antibiotic represents the number of resistant isolates to that antibiotic.**
