# Supplementary material for: Promising FDA-approved drugs with efflux pump inhibitory activities against clinical isolates of Staphylococcus aureus
Source: PLoS One. 2022 Jul 29;17(7):e0272417. doi: 10.1371/journal.pone.0272417 (PMC9337675; doi:10.1371/journal.pone.0272417)
Supplement: S6 Table — No., the isolate number; P, positive efflux isolate; N, negative efflux isolate; I, intermediate efflux isolate; W, isolated from wound; U, isolated from urine; B, isolated from burn; E, isolated from endotracheal aspirate; S, isolated from sputum; +, gene present; -, gene absent. (DOCX) [file pone.0272417.s006.docx]

**Supplementary Table 6. Results of EtBr Cart-Wheel method and the prevalence of efflux resistance genes in the selected isolates (n=72)**

| **Isolate No.** | **Isolate code** | **MC of EtBr produced F** | **EtBr-CW result** | **Genotypic investigation of efflux genes by PCR** | | | |
| --- | --- | --- | --- | --- | --- | --- | --- |
|  |  |  |  | ***tet*K** | ***nor*A** | ***fex*A** | ***msr*A** |
| **1** | **E 189** | 4 | P | + | + | + | **−** |
| **2** | **B 951** | 1 | N | + | + | **−** | **−** |
| **3** | **B 866** | 3 | P | + | + | **−** | **−** |
| **4** | **B 3** | 3 | P | + | + | + | **−** |
| **5** | **B 4** | 1 | N | + | + | **−** | **−** |
| **6** | **B 15** | 1 | N | + | + | **−** | **−** |
| **7** | **B 21** | 3 | P | + | + | **−** | **−** |
| **8** | **B 39** | 2 | I | + | + | **−** | **−** |
| **9** | **B 40** | 2 | I | + | + | **−** | **−** |
| **10** | **B 48** | 3 | P | + | + | **−** | **−** |
| **11** | **B 46** | 2 | I | + | + | **−** | **−** |
| **12** | **B 50** | 3 | P | + | + | + | **−** |
| **13** | **B 72** | 1 | N | + | + | + | **−** |
| **14** | **B 97** | 2 | I | + | + | + | **−** |
| **15** | **B 776** | 2 | I | + | + | + | **−** |
| **16** | **B 783** | 2 | I | + | + | **−** | **−** |
| **17** | **B 789** | 1 | N | + | + | + | **−** |
| **18** | **U 426** | 1 | N | + | + | **−** | **−** |
| **19** | **W 822** | 2 | I | + | + | + | **−** |
| **20** | **W 823** | 3 | P | + | + | **−** | **−** |
| **21** | **W 871** | 4 | P | + | + | **−** | **−** |
| **22** | **W 898** | 3 | P | + | + | **−** | **−** |
| **23** | **W 429** | 2 | I | + | + | + | **−** |
| **24** | **S 437** | 2 | I | + | + | **−** | **−** |
| **25** | **S 417** | 3 | P | **−** | + | **−** | **−** |
| **26** | **B 864** | 2 | I | + | + | + | **−** |
| **27** | **B 868** | 3 | P | + | + | + | **−** |
| **28** | **B 23** | 1 | N | **−** | + | + | **−** |
| **29** | **B 20** | 1 | N | **−** | + | **−** | **−** |
| **30** | **B 14** | 1 | N | + | + | **−** | **−** |
| **31** | **B 787** | 1 | N | + | + | + | **−** |
| **32** | **U 418** | 1 | N | **−** | + | **−** | **−** |
| **33** | **W 904** | 2 | I | + | + | **−** | **−** |
| **34** | **W 428** | 1 | N | + | + | **−** | **−** |
| **35** | **W 881** | 1 | N | + | + | **−** | **−** |
| **36** | **W 869** | 1 | N | + | + | **−** | **−** |
| **37** | **W 877** | 1 | N | + | + | **−** | **−** |
| **38** | **W 820** | 3 | P | + | + | **−** | **−** |
| **39** | **B 975** | 1 | N | + | + | **−** | + |
| **40** | **W 436** | 2 | I | + | + | **−** | **−** |
| **41** | **B 767** | 2 | I | + | + | **−** | **−** |
| **42** | **B 950** | 2 | I | + | + | **−** | **−** |
| **43** | **B 856** | 1 | N | + | + | **−** | **−** |
| **44** | **B 774** | 4 | P | + | + | + | **−** |
| **45** | **B 786** | 3 | P | + | + | + | **−** |
| **46** | **W 873** | 2 | I | + | + | **−** | **−** |

**Supplementary Table 6. Continued**

| **Isolate No.** | **Isolate code** | **MC of EtBr produced F** | **EtBr-CW Result** | **Genotypic investigation of efflux genes by PCR** | | | |
| --- | --- | --- | --- | --- | --- | --- | --- |
|  |  |  |  | ***tet*K** | ***nor*A** | ***fex*A** | ***msr*A** |
| **47** | **W 887** | 1 | N | + | + | **−** | **−** |
| **48** | **W 446** | 2 | I | **−** | + | **−** | **−** |
| **49** | **E 444** | 2 | I | **−** | + | + | **−** |
| **50** | **W 914** | 3 | P | + | + | + | **−** |
| **51** | **B 856** | 2 | I | + | + | **−** | **−** |
| **52** | **B 956** | 2 | I | + | + | **−** | **−** |
| **53** | **B 974** | 4 | P | + | + | **−** | **−** |
| **54** | **B 791** | 1 | N | + | + | **−** | **−** |
| **55** | **W 916** | 2 | I | + | + | **−** | **−** |
| **56** | **W 915** | 2 | I | + | + | **−** | **−** |
| **57** | **B 14** | 1 | N | **−** | + | **−** | **−** |
| **58** | **B 865** | 2 | I | **−** | + | + | **−** |
| **59** | **B 84** | 3 | P | + | + | **−** | + |
| **60** | **B 26** | 2 | I | **−** | **−** | **−** | + |
| **61** | **B 771** | 1 | N | **−** | **−** | + | **−** |
| **62** | **U 399** | 2 | I | + | + | **−** | **−** |
| **63** | **W 752** | 1 | N | **−** | + | **−** | + |
| **64** | **B 31** | 2 | I | **−** | + | **−** | + |
| **65** | **B 969** | 2 | I | + | **−** | **−** | **−** |
| **66** | **W 628** | 3 | P | + | + | + | **−** |
| **67** | **W 629** | 2 | I | + | + | **−** | **−** |
| **68** | **W 885** | 1 | N | + | + | **−** | **−** |
| **69** | **U 419** | 1 | N | **−** | + | **−** | **−** |
| **70** | **W 824** | 2 | I | **−** | **−** | **−** | **−** |
| **71** | **W 527** | 1 | N | **−** | **−** | **−** | **−** |
| **72** | **W 380** | 2 | I | **−** | **−** | **−** | **−** |
| **73** | **St.** | 0.5 | N | **−** | **−** | **−** | **−** |

**No., the isolate number; St., the standard strain, *S*. *aureus* ATCC 25923; P, positive efflux isolate; N, negative efflux isolate; I, intermediate efflux isolate; W, isolated from wound; U, isolated from urine; B, isolated from burn; E, isolated from endotracheal aspirate; S, isolated from sputum; +, gene present; -, gene absent**.
