# Supplementary material for: Promising FDA-approved drugs with efflux pump inhibitory activities against clinical isolates of Staphylococcus aureus
Source: PLoS One. 2022 Jul 29;17(7):e0272417. doi: 10.1371/journal.pone.0272417 (PMC9337675; doi:10.1371/journal.pone.0272417)
Supplement: S7 Table — No., isolate number; (+), positive for the gene; (-), negative for the gene; EtBrCW, EtBr Cart-Wheel test. (DOCX) [file pone.0272417.s007.docx]

**Supplementary Table 7.** **The profile of the selected isolates (n=26) for further analysis of efflux and testing the activity of tested drugs**

| **No.** | **Isolate code** | ***tet*K** | ***nor*A** | ***fex*A** | ***msr*A** | **EtBrCW result** |
| --- | --- | --- | --- | --- | --- | --- |
| **1** | **E 189** | + | + | + | **−** | Positive |
| **2** | **B 866** | + | + | **−** | **−** | Positive |
| **3** | **B 3** | + | + | + | **−** | Positive |
| **4** | **B 50** | + | + | + | **−** | Positive |
| **5** | **W 898** | + | + | **−** | **−** | Positive |
| **6** | **S 417** | **−** | + | **−** | **−** | Positive |
| **7** | **B 868** | + | + | + | **−** | Positive |
| **8** | **B 774** | + | + | + | **−** | Positive |
| **9** | **B 786** | + | + | + | **−** | Positive |
| **10** | **W 914** | + | + | + | **−** | Intermediate |
| **11** | **W 628** | + | + | + | **−** | Positive |
| **12** | **B 97** | + | + | + | **−** | Intermediate |
| **13** | **776** | + | + | + | **−** | Intermediate |
| **14** | **B 864** | + | + | + | **−** | Intermediate |
| **15** | **B 84** | + | + | **−** | + | Positive |
| **16** | **B 21** | + | + | **−** | **−** | Positive |
| **17** | **B 783** | + | + | **−** | **−** | Intermediate |
| **18** | **W 823** | + | + | **−** | **−** | Positive |
| **19** | **W 871** | + | + | **−** | **−** | Positive |
| **20** | **W 820** | + | + | **−** | **−** | Positive |
| **21** | **B 48** | + | + | **−** | **−** | Positive |
| **22** | **W 873** | + | + | **−** | **−** | Intermediate |
| **23** | **E 444** | **−** | + | + | **−** | Intermediate |
| **24** | **B 31** | **−** | + | **−** | + | Intermediate |
| **25** | **W 446** | **−** | + | **−** | **−** | Intermediate |
| **26** | **B 26** | − | − | **−** | + | Intermediate |
| **27** | **St.** | **−** | **−** | **−** | **−** | Negative |

**No., isolate number; St., *S. aureus* ATCC 25923; (+), positive for the gene; (-), negative for the gene; EtBrCW, EtBr Cart-Wheel test.**
