# Supplementary material for: Promising FDA-approved drugs with efflux pump inhibitory activities against clinical isolates of Staphylococcus aureus
Source: PLoS One. 2022 Jul 29;17(7):e0272417. doi: 10.1371/journal.pone.0272417 (PMC9337675; doi:10.1371/journal.pone.0272417)
Supplement: S13 Table — Conc., concentration; AO, acridine orange; +, fluorescence; ++, high fluorescence; +++, very high fluorescence. (DOCX) [file pone.0272417.s013.docx]

**Supplementary Table 13. The inhibitory effect of metformin on the efflux activity of isolates by Cart-Wheel method using acridine orange**

| **Isolate No.** | **Isolate code** | **Efflux activity** | **Conc. of AO (mg/L) and degree of fluorescence produced** | | | | | |
| --- | --- | --- | --- | --- | --- | --- | --- | --- |
|  |  |  | **0.5** | **1** | **2** | **5** | **10** | **20** |
| **1** | **E 189** | Negative | - | + | + | + | ++ | ++ |
| **2** | **B 866** | Negative | - | + | + | ++ | ++ | +++ |
| **3** | **B 3** | Negative | - | + | + | + | ++ | ++ |
| **4** | **B 50** | Negative | - | + | + | + | ++ | ++ |
| **5** | **W 898** | Negative | - | + | + | ++ | ++ | +++ |
| **6** | **S 417** | Negative | - | + | + | ++ | ++ | +++ |
| **7** | **B 868** | Negative | - | + | + | + | ++ | ++ |
| **8** | **B 774** | Negative | - | + | + | + | ++ | ++ |
| **9** | **B 786** | Negative | - | + | + | + | ++ | ++ |
| **10** | **W 914** | Negative | - | + | + | ++ | ++ | +++ |
| **11** | **W 628** | Negative | - | + | + | ++ | ++ | +++ |
| **12** | **B 97** | Negative | - | + | + | + | ++ | ++ |
| **13** | **B 776** | Negative | - | + | + | + | ++ | ++ |
| **14** | **B 864** | Negative | - | + | + | + | ++ | ++ |
| **15** | **B 84** | Negative | - | + | + | + | ++ | ++ |
| **16** | **B 21** | Negative | - | + | + | + | ++ | ++ |
| **17** | **B 783** | Negative | - | + | + | + | ++ | ++ |
| **18** | **W 823** | Negative | - | + | + | + | ++ | ++ |
| **19** | **W 871** | Negative | - | + | + | + | ++ | ++ |
| **20** | **W 820** | Negative | - | + | + | + | ++ | ++ |
| **21** | **B 48** | Negative | - | + | + | + | ++ | ++ |
| **22** | **W 873** | Negative | - | + | + | + | ++ | ++ |
| **23** | **E 444** | Negative | - | + | + | + | ++ | ++ |
| **24** | **B 31** | Negative | - | + | + | + | ++ | ++ |
| **25** | **W 446** | Negative | - | + | + | + | ++ | ++ |
| **26** | **B 26** | Negative | - | + | + | + | ++ | ++ |

**Conc., concentration; AO, acridine orange; -, no fluorescence; +, fluorescence; ++, high fluorescence; +++, very high fluorescence.**
