# Supplementary material for: Promising FDA-approved drugs with efflux pump inhibitory activities against clinical isolates of Staphylococcus aureus
Source: PLoS One. 2022 Jul 29;17(7):e0272417. doi: 10.1371/journal.pone.0272417 (PMC9337675; doi:10.1371/journal.pone.0272417)
Supplement: S14 Table — VP, verapamil; GTN, glyceryl trinitrate; D, domperidone; MF, metformin; DF, diclofenac sodium; ND, no decrease in MIC. (DOCX) [file pone.0272417.s014.docx]

**Supplementary Table 14. MICs (µg/ml) of ciprofloxacin (CIP) and chloramphenicol (C) alone and in the presence of the tested compounds**

| **Isolate code** | **CIP** | **CIP _VP_** | **CIP _DF_** | **CIP _GTN_** | **CIP _D_** | **CIP _MF_** | **C** | **C _VP_** | **C _DF_** | **C _GTN_** | **C _D_** | **C _MF_** |
| --- | --- | --- | --- | --- | --- | --- | --- | --- | --- | --- | --- | --- |
| **E 189** | 256 | 16 (16) | 4 (64) | 16 (16) | 4 (64) | 8 (32) | 512 | 32 (16) | 16 (32) | 16 (32) | 16 (32) | 32 (16) |
| **B 866** | 256 | 8 (32) | 2 (128) | 2 (128) | 2 (128) | 64 (4) | 1024 | 128 (8) | 128 (8) | 128 (8) | 256 (4) | 256 (4) |
| **B 3** | 128 | 32 (4) | 2 (64) | 8 (16) | 2 (64) | 64 (2) | 1024 | 32 (32) | 4 (256) | 8 (128) | 16 (64) | 4 (256) |
| **B 50** | 256 | 32 (8) | 4 (64) | 16 (16) | 2 (128) | 64 (4) | 1024 | 128 (8) | 32 (32) | 32 (32) | 256 (4) | 256 (4) |
| **W 898** | 256 | 32 (8) | 8 (32) | 16 (16) | 8 (32) | 64 (4) | 16 | 1 (16) | 1 (16) | 0.5 (32) | 4 (4) | 2 (8) |
| **S 417** | 256 | 32 (8) | 2 (128) | 8 (32) | 2 (128) | 32 (8) | 128 | 8 (16) | 2 (64) | 2 (64) | 2 (64) | 16 (8) |
| **B 868** | 256 | 8 (32) | 2 (128) | 8 (32) | 2 (128) | 8 (32) | 1024 | 128 (8) | 8 (128) | 16 (64) | 16 (64) | 256 (4) |
| **B 774** | 256 | 16 (16) | 16 (16) | 16 (16) | 16 (16) | 32 (8) | 1024 | 8 (128) | 2 (512) | 2 (512) | 4 (256) | 32 (32) |
| **B 786** | 256 | 8 (32) | 8 (32) | 16 (16) | 8 (32) | 32 (8) | 1024 | 16 (64) | 8 (128) | 8 (128) | 8 (128) | 64 (16) |
| **W 914** | 64 | 16 (4) | 4 (16) | 8 (8) | 4 (16) | 16 (4) | 1024 | 32 (32) | 2 (512) | 2 (512) | 2 (512) | 256 (4) |
| **W 628** | 64 | 8 (8) | 4 (16) | 4 (16) | 4 (16) | 16 (4) | 1024 | 64 (16) | 4 (256) | 8 (128) | 4 (256) | 512 (2) |
| **B 97** | 256 | 16 (16) | 16 (16) | 64 (4) | 16 (16) | 256 (ND) | 1024 | 64 (16) | 4 (256) | 4 (256) | 4 (256) | 512 (2) |
| **B 776** | 256 | 16 (16) | 4 (64) | 16 (16) | 4 (64) | 4 (64) | 1024 | 32 (32) | 8 (128) | 8 (128) | 8 (128) | 64 (16) |
| **B 864** | 128 | 32 (4) | 8 (16) | 2 (64) | 4 (32) | 64 (2) | 1024 | 64 (16) | 4 (256) | 8 (128) | 8 (128) | 256 (4) |
| **B 84** | 64 | 16 (4) | 4 (16) | 16 (4) | 4 (16) | 32 (2) | 0.5 | 0.5 (ND) | 0.5 (ND) | 0.5 (ND) | 0.5 (ND) | 0.5 (ND) |
| **B 21** | 256 | 32 (8) | 16 (16) | 32 (8) | 32 (8) | 64 (4) | 1024 | 64 (16) | 16 (64) | 16 (64) | 16 (64) | 128 (8) |
| **B 783** | 512 | 32 (16) | 32 (16) | 32 (16) | 32 (16) | 64 (8) | 1024 | 128 (8) | 16 (64) | 16 (64) | 16 (64) | 512 (2) |
| **W 823** | 256 | 32 (8) | 4 (64) | 16 (16) | 8 (32) | 64 (4) | 1024 | 64 (16) | 16 (64) | 32 (32) | 16 (64) | 128 (8) |
| **W 871** | 256 | 32 (8) | 2 (128) | 8 (32) | 8 (32) | 64 (4) | 1024 | 16 (64) | 4 (256) | 8 (128) | 8 (128) | 256 (4) |
| **W 820** | 256 | 16 (16) | 4 (64) | 8 (32) | 8 (32) | 32 (8) | 0.5 | 0.5 (ND) | 0.5 (ND) | 0.5 (ND) | 0.5 (ND) | 0.5 (ND) |
| **B 48** | 256 | 8 (32) | 2 (128) | 2 (128) | 2 (128) | 64 (4) | 1024 | 64 (16) | 4 (256) | 4 (256) | 4 (256) | 256 (4) |
| **W 873** | 256 | 32 (8) | 8 (32) | 32 (8) | 16 (16) | 32 (8) | 512 | 32 (16) | 8 (64) | 2 (256) | 2 (256) | 256 (2) |
| **E 444** | 16 | 2 (8) | 0.5 (32) | 2 (8) | 0.5 (32) | 4 (4) | 512 | 32 (16) | 8 (64) | 4 (128) | 4 (128) | 32 (16) |
| **B 31** | 64 | 16 (4) | 4 (16) | 4 (16) | 4 (16) | 16 (4) | 0.5 | 0.5 (ND) | 0.5 (ND) | 0.5 (ND) | 0.5 (ND) | 0.5 (ND) |
| **W 446** | 128 | 32 (4) | 32 (4) | 32 (4) | 32 (4) | 64 (2) | 0.5 | 0.5 (ND) | 0.5 (ND) | 0.5 (ND) | 0.5 (ND) | 0.5 (ND) |
| **B 26** | 0.5 | 0.5 (ND) | 0.5 (ND) | 0.5 (ND) | 0.5 (ND) | 0.5 (ND) | 256 | 8 (32) | 4 (64) | 2 (128) | 2 (128) | 16 (16) |

**VP, verapamil; GTN, glyceryl trinitrate; D, domperidone; MF, metformin; DF, diclofenac sodium; ND, no decrease in MIC.**
