# Supplementary figures and images for: Promising FDA-approved drugs with efflux pump inhibitory activities against clinical isolates of Staphylococcus aureus
Source: PLoS One. 2022 Jul 29;17(7):e0272417. doi: 10.1371/journal.pone.0272417 (PMC9337675; doi:10.1371/journal.pone.0272417)

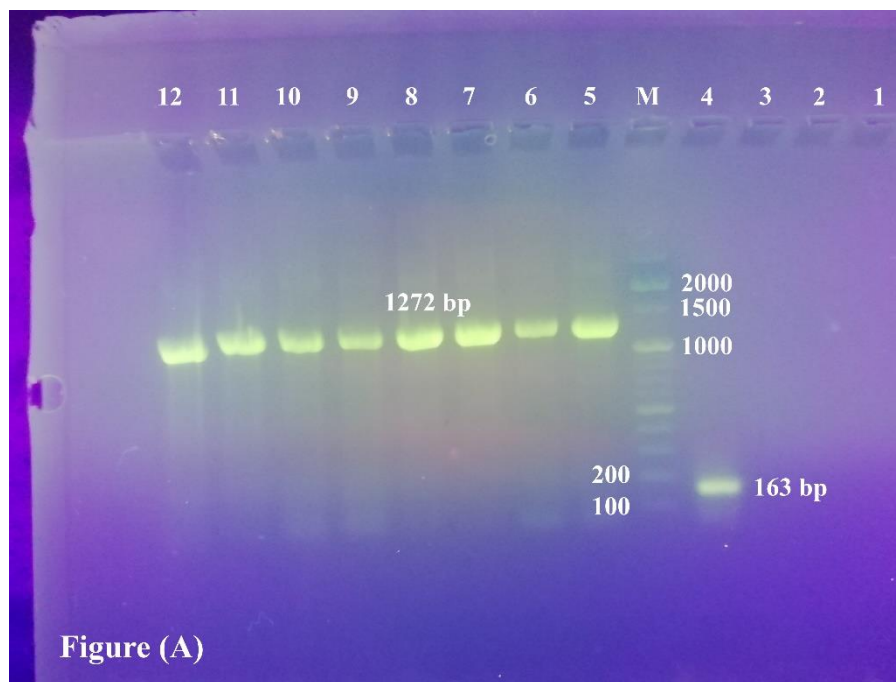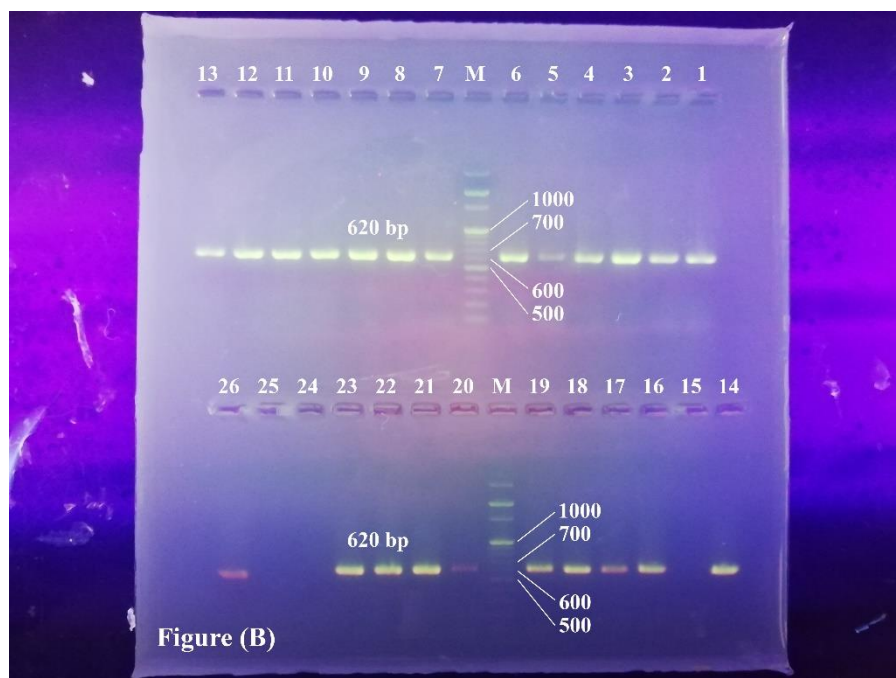

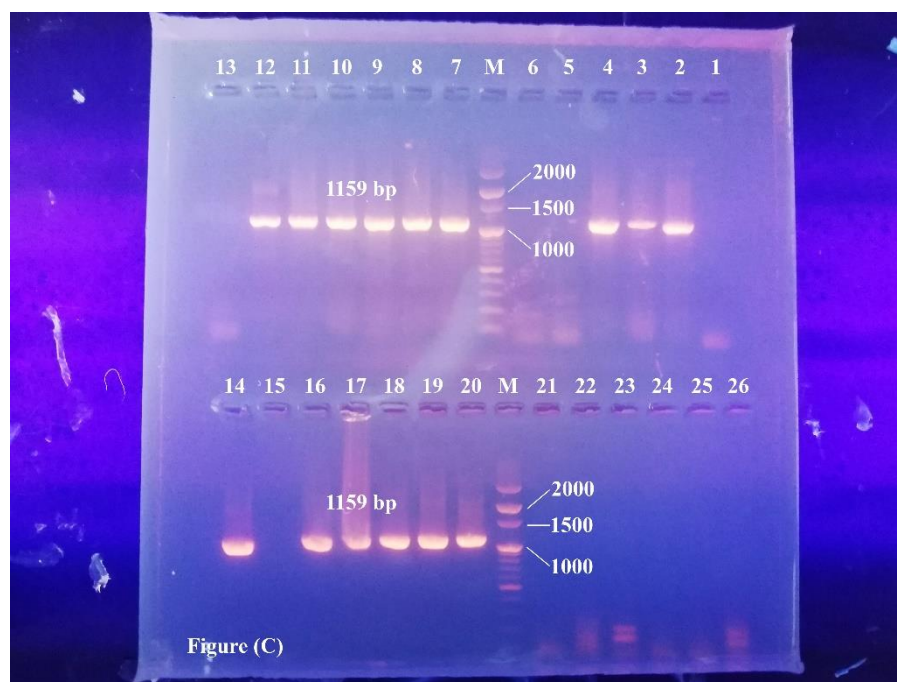

Supplement: S1 Raw images — (A) Original uncropped and unadjusted image of fexA and msrA genes. M; DNA ladder marker (3000 bp), Lanes 5–12; fexA gene (1272 bp), Lane 4; msrA gene (163 bp) and lanes 1–3; negative msrA isolates. (B) Original uncropped and unadjusted image of norA gene. M; DNA ladder marker (3000 bp), Lanes 1–14, 16–23, 26; norA gene (620 bp) and Lanes 15, 24, 25; negative norA isolates. (C) Original uncropped and unadjusted image of tetK gene. M; DNA ladder marker (3000 bp), Lanes 2–4, 7–12, 14, 16–20; tetK gene (1159 bp), Lanes 1, 5, 6, 13, 15; contained negative tetK isolates and Lanes 21–26; negative results of another detected gene. (PDF) [file pone.0272417.s017.pdf]
